# Neural spatio-temporal patterns of information processing related to cognitive conflict and correct or false recognitions

**DOI:** 10.1038/s41598-022-09141-9

**Published:** 2022-03-28

**Authors:** Romuald A. Janik, Igor T. Podolak, Łukasz Struski, Anna Ceglarek, Koryna Lewandowska, Barbara Sikora-Wachowicz, Tadeusz Marek, Magdalena Fafrowicz

**Affiliations:** 1 Institute of Theoretical Physics, Jagiellonian University, Kraków, 30-348 Poland; 2 Group of Machine Learning GMUM, Faculty of Mathematics and Computer Science, Jagiellonian University, Kraków, 30-348 Poland; 3 Department of Cognitive Neuroscience and Neuroergonomics, Institute of Applied Psychology, Jagiellonian University, Kraków, 30-348 Poland

**Keywords:** Cognitive neuroscience, Computational neuroscience

## Abstract

Using a visual short-term memory task and employing a new methodological approach, we analyzed neural responses from the perspective of the conflict level and correctness/erroneous over a longer time window. Sixty-five participants performed the short-term memory task in the fMRI scanner. We explore neural spatio-temporal patterns of information processing in the context of correct or erroneous response and high or low level of cognitive conflict using classical fMRI analysis, surface-based cortical data, temporal analysis of interpolated mean activations, and machine learning classifiers. Our results provide evidence that information processing dynamics during the retrieval process vary depending on the correct or false recognition—for stimuli inducing a high level of cognitive conflict and erroneous response, information processing is prolonged. The observed phenomenon may be interpreted as the manifestation of the brain’s preparation for future goal-directed action.

## Introduction

Cognitive control is a neuropsychological construct, describing the process in which information processing and behaviour vary from moment to moment, adapting to current goals and alterations of the environment. This process is guided mostly by the prefrontal cortex and its activation is depending on the demand required to better perform or complete a task. The essential function of cognitive control is conflict monitoring. Conflict monitoring includes two components: the monitoring component, which evaluates the degree of conflict, and the second one—control adaptation, which adjusts attentional filters to the task demands^1^. The link between the two components has been confirmed in studies using congruent and incongruent stimuli consecutively, which demonstrated that increased conflict monitoring is associated with an increased size of sequential congruence effect (index of control adaptation)^2,3^. According to the conflict-monitoring theory, the detected conflict or error triggers a negative affecting signal^4,5^, which drives the control adaptation^6,7^. Some researchers assumed that errors and conflicts can be considered as the same events^8^. However, recent error-related negativity (ERN) studies suggested that neural networks of error and conflict monitoring are disparate^9^.

The usually chosen pivotal tasks for studying the cognitive conflict are Stroop, Simon, or Flanker tasks, where the incongruent trials cause the interference in processing and require inhibition and reversion of the motor reaction/response. Nevertheless, some experimental paradigms investigating memory also invoke cognitive conflict of varying intensity. In the current study, a popular paradigm for investigating false memories formation—the Deese–Roediger–McDermott (DRM) paradigm—was applied^10,11^. In the original version of the DRM task, the students were asked to recall a previously read list of twelve words related to the not previously presented critical lure^10^. It turned out that the participants recalled more often the lure-word among related words. Such false recognition effects have been demonstrated also for visual stimuli (abstract shapes)^12^. Therefore, the DRM paradigm was incrementally modified to investigate false recognition with other material types (semantic, phonological, visual) as well as other memory types (working, long-term, episodic etc.). Neuroimaging studies using the DRM paradigm indicate the greatest activations in the prefrontal and visual regions in relation to false memories (for a review, see: REF^13^). Atkins and Reuter-Lorenz with semantic interference in short-term memory revealed increased dorsolateral prefrontal cortex and fusiform gyrus activations associated with the correct rejection of related lure and true recognition, respectively^14^. Some research also demonstrated neural mechanisms of true and false recognition with the use of visual stimuli (including abstract objects). Slotnick and Schacter, using this type of memoranda, showed activations of prefrontal, parietal and visual regions correlated with true recognition as well as frontal, insular, and temporal cortices— with false recognition^15^. Likewise, Garoff-Eaton indicated prefrontal, parietal and temporal cortices associated with both true and false related recognition^16^. According to our knowledge, there are no studies that investigate the neural response in the DRM paradigm from the point of view of cognitive conflict of varying intensity.

To provide a detailed insight into spatio-temporal patterns of information processing related to correct and erroneous responses and different levels of cognitive conflict, we analyse four types of responses: correct recognition of positive probes (POScorr), correct rejection of lure probes (LURcorr), false recognition of lure probes (LURfalse) and correct rejection of negative probes (NEGcorr) in two types of contrasts (POScorr–LURfalse and NEGcorr–LURcorr). Traditionally, researchers who investigate the cognitive conflict using fMRI techniques have addressed this aspect employing the General Linear Model (GLM) method of analysis^17,18^. With the recent methodological advances occurring, the new perspectives to investigate the spatio-temporal dynamics of information processing, which is not possible with classical methods. The standard GLM analysis allows identifying regions whose activations are statistically relevant for a particular contrast. The conventional GLM setup, however, does not yield more fine-grained information about the *differences* in the temporal structure of the responses for different events and/or different regions.

In this study, we employed the classical fMRI analysis method and two non-standard methods of fMRI analysis: machine learning methods and interpolated mean signals analysis on volumetric and surface-based data. The machine learning classifiers trained to distinguish events based on data from a particular time repetition (TR) provide to assess the amount of information contained in the brain at a particular time after retrieval, which allows distinguishing the correct and false responses. This gives us the first indication that there is quite a lot of relevant activation rather late after the retrieval event, which motivates our subsequent, more detailed study. As the area under curve (AUC) of the machine learning classifiers can be understood as measuring available information for distinguishing two events, we use them to assess whether surface based registration of cortical data is better in this respect than the standard volumetric analysis. Then, the analysis of mean interpolated signals for each event, allows us to identify relative temporal delays in processing between the events as well as isolate regions which exhibit significantly late activations. Finally, our analysis of Shapley values gives a complementary picture using a state-of-the-art machine learning methodology.

The main goal of this paper is to investigate the neural mechanism of changing cognitive demand in short-term memory. It is the first attempt of using surface-based data with short-term false memory research. We investigate the spatio-temporal features of information processing related to erroneous and correct responses and varying intensity of cognitive conflict with the use of new methods of fMRI data analysis.

## Results

### Behavioural results

The general linear model (GLM) with accuracy and reaction times as dependent variables and probe types as fixed factor was performed (for description of probe types, see “Methods” section). In case of accuracy, the probe type was significant ($F(1,4)=445$, $p<0.0001$, $\eta _{p}^{2}=0.848$). The HSD Tukey’s post-hoc tests revealed differences between all probe types (p < 0.0001). For the reaction times (RTs), the probe type was also significant ($F(1,4)=59.29$, $p<0.0001$, $\eta _{p}^{2}=0.426$). The HSD Tukey’s post-hoc tests revealed differences between all probe types ($p<0.0001$) except pairs: POScorr–LURcorr and LURcorr–LURfalse. The descriptive statistics on accuracy and RTs are presented in Fig. A3 (see *Supplementary Information*). Post-error slowing (PES) was not confirmed. The t tests between reaction times for all trials and trials after erroneous responses for positive probes ($T(128)=0.634$, $p=0.527$) and for lure probes ($T(128)=0.450$, $p=0.653$) were not significant.

### GLM results

The GLM analysis with elongated duration of events (see “Methods” for details) was performed to validate the results on surface-based data. The group analysis revealed significant clusters only for the contrasts POScorr>LURfalse and LURfalse>POScorr. For other contrasts, no significant results were observed.

- POScorr > LURfalse at retrieval (section **a** of Fig. A1, *Supplementary Information*) left middle occipital gyrus ($T=5.77$, $k=75$, $p(FDR\; corr.)=0.020$), left and right precuneus ($T=5.17$, $k=379$, $p(FDR\; corr.)<0.001$).
- LURfalse > POScorr at retrieval (section **b** on Fig. A1, *Supplementary Information*) right middle frontal gyrus ($T=5.22$, $k=156$, $p(FDR\;corr.)=0.001$), right superior frontal gyrus medial part ($T=5.18$, $k=394$, $p(FDR\; corr.)<0.001$).

### Motivation for using surface-based HCP style data

**Figure 1:**
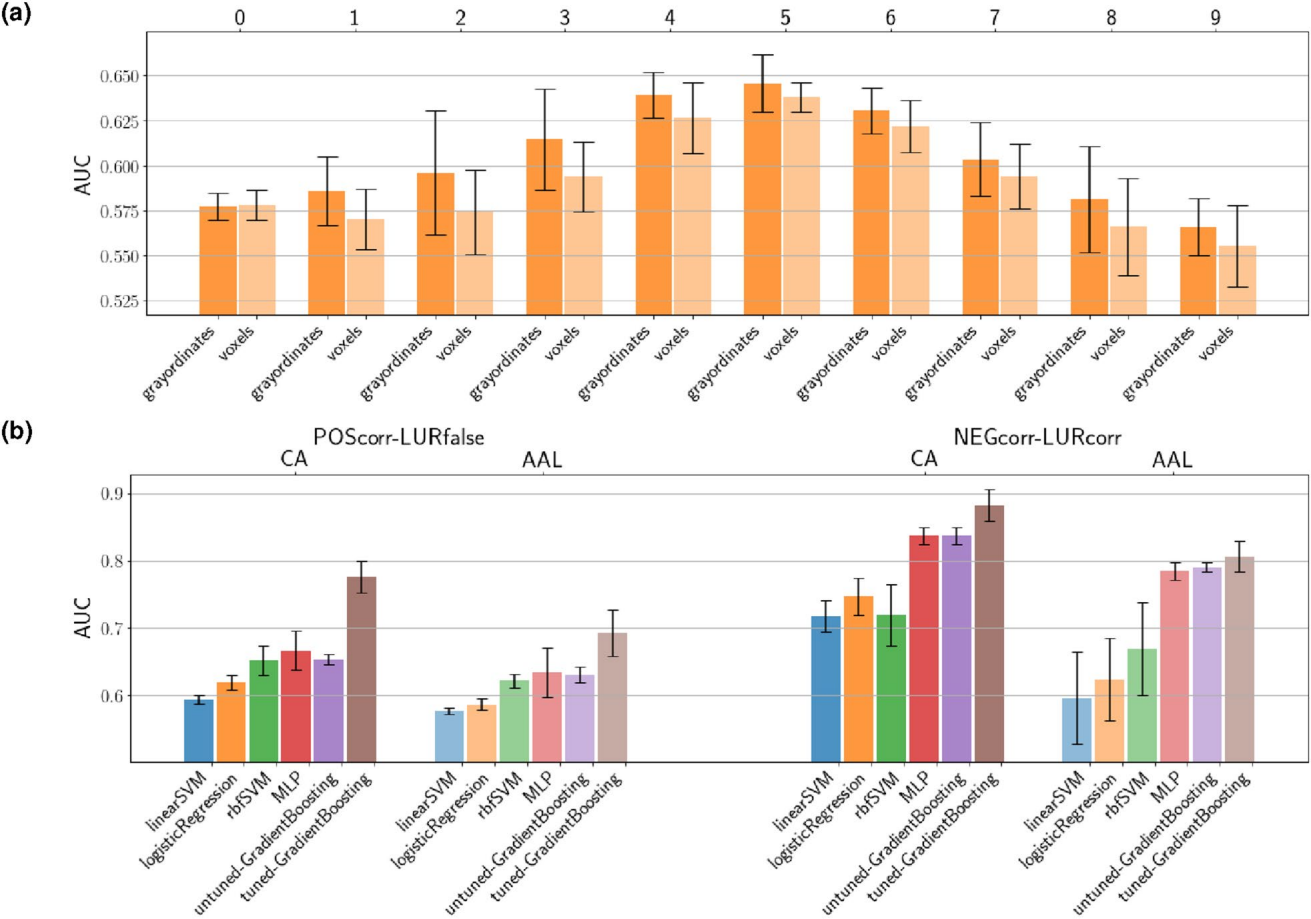
The mean of AUC for 5-fold cross-validation of different classification methods. (**a**) Results of logistic regression classifier for POScorr–LURfalse at time 0–9 TR (upper horizontal axis) post retrieval event for both surface and volumetric data (bottom horizontal axis). All data was normalized to have zero mean and unit standard deviation. (**b**) Results for two type of data: *Cole–Anticevic* (CA) and *Automated Anatomical Labelling* (AAL). We consider two classification problems *POScorr–LURfalse*, *NEGcorr–LURcorr* and five classifiers: *logistic regression*, *linear SVM*, *rbf SVM*, *MLP* (deep classifier), and the gradient boosting of decision trees^21^—two cases: *untuned* GradientBoosting, *tuned* GradientBoosting. The tuning was performed by computing first Shapley contribution values for all features and computing models with only the most contributing features (see Shapley values in “Methods” and Fig. A4, *Supplementary Information*, for AUC values of models with different number of features). About 10–15% of features proved to be satisfactory.

In contrast to conventional volumetric fMRI data, this work uses, to a large extent, surface-based data in the form pioneered by the Human Connectome Project^19^. The key difference is that the cortex is represented by a 2D surface mesh, while the subcortical anatomical structures are represented through voxels. The vertices and voxels are then collectively referred to as *grayordinates*. The mapping of the cortex to the 2D mesh incorporates individual folding patterns so that the MNI coordinates of a particular vertex differ between participants. On the other hand, the mapping aims to maximize the *anatomical/functional* identification of a given vertex across different participants and thus should enhance the quality of inter-subject analysis w.r.t. conventional volumetric data. A quantitative comparison^20^ indicates that this is indeed the case. As shown in Fig. 1 (for details see “Methods”) the superiority of surface data also applies in our setting. Let us also note that the classification accuracy for the contrast NEGcorr–LURcorr are noticeably higher than for the contrast POScorr–LURfalse, which indicates that the distinction in the brain activity between the latter two events is much more subtle.

Let us now return to the distinctive pattern of the classifier performance as a function of time after the retrieval event seen in Fig. 1a. It is striking that the best performance is obtained as late as 5 TR after the retrieval event, with 6 TR being also quite high. This indicates that the brain activations are significantly different between the POScorr or LURfalse even quite late after the retrieval event. We shall study the details of the brain’s temporal response in the following section.

### Temporal structure of the response to a retrieval event

The subject’s neural response associated with a retrieval event has a non-trivial temporal profile, which depends on whether the subject gave a false answer to a lure probe or a true answer to a positive probe. Beside the correctness (POScorr–LURfalse), the high and low (NEGcorr–LURcorr) level of cognitive conflict is considered. In order to uncover the temporal structure, we have to go beyond just associating a standard haemodynamic response function (HRF) to the retrieval event, so we adopt a different methodology, somewhat analogous to event-related-potentials (ERP) in EEG, but of course on a completely different timescale.

After appropriately normalizing the individual fMRI signals (see “Methods”) and projecting to the Cole-Anticevic parcellation, we take the mean signal over all trials with a given response (POScorr, LURfalse, NEGcorr, and LURcorr), temporally locked to the TR frame with the retrieval event. In this way, we may expect noise and neural processes unrelated to the event and the specific response to cancel out. In order to ascertain the statistical relevance of the particular regions, we adopt permutation tests to account for False Discovery Rate and use bootstrap for estimating statistical errors for the introduced observables. The observables of interest are defined using a spline interpolation of the mean signals (see “Methods” for details). The interpolated signal is also used for visualization.

#### Early stage of the neural response for the POScorr–LURfalse contrast

Among the 718 regions, we first select regions whose (interpolated) mean neural response to both events (i.e., POScorr or LURfalse) has a local maximum in the period 1–5 TR after the retrieval event. We then quantify their sensitivity to the POScorr–LURfalse contrast by measuring the area between the respective mean responses in the period 0–5 TR. In this way, we obtain 33 statistically relevant regions for the early stage of processing (see “Methods”). The regions are listed in Table A1 (*Supplementary Information*).

**Figure 2:**
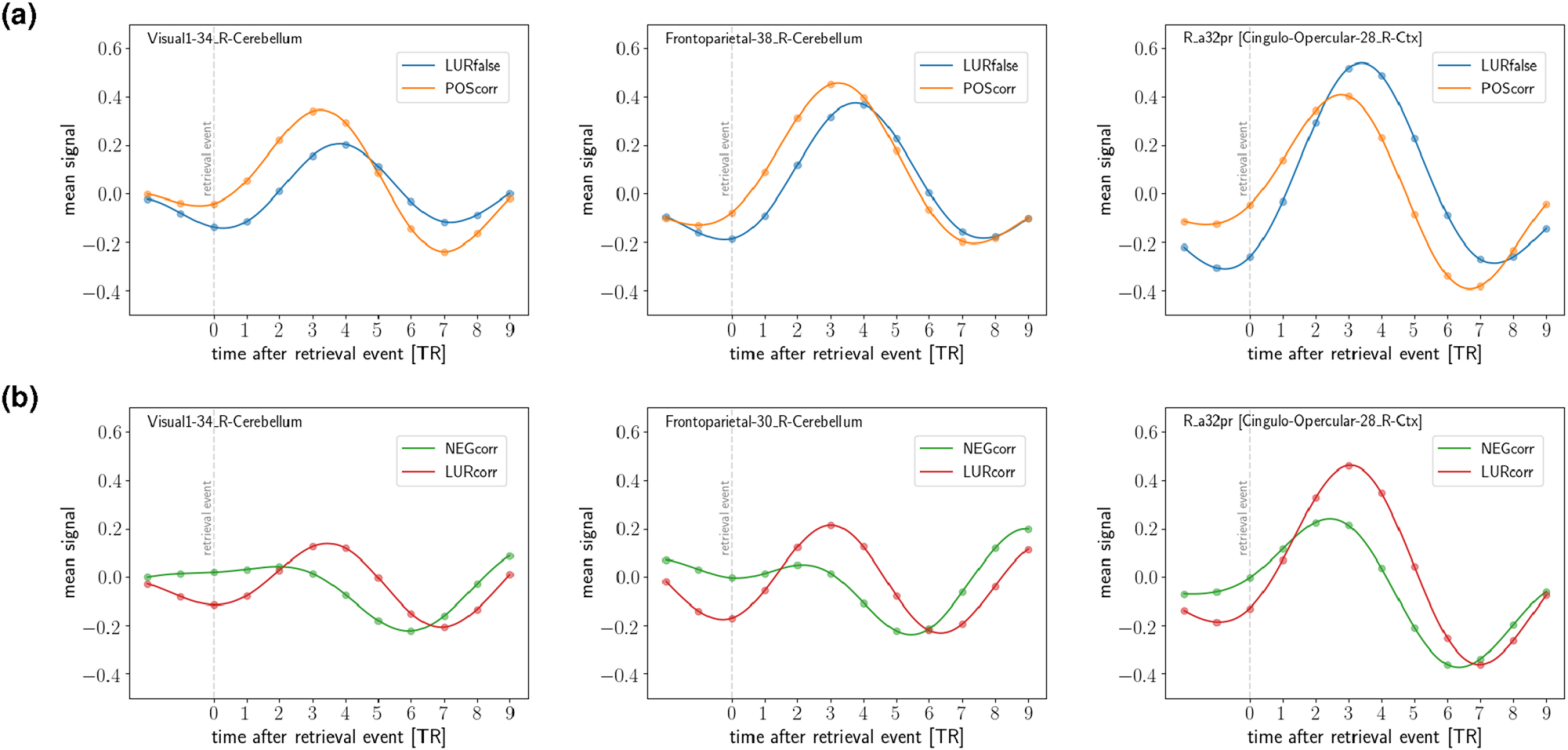
Differences in the course of the neural response for two contrasts (POScorr–LURfalse and NEGcorr–LURcorr). (**a**) Mean signals temporally locked to the retrieval event for POScorr–LURfalse for three selected subcortical and cortical regions. (**b**) Mean signals temporally locked to the retrieval event for lower (NEGcorr) and higher (LURcorr) levels of cognitive conflict for three selected regions, with maxima in the early time window.

In Fig. 2a, we show representative examples of regions which exhibit a temporally differentiated response to the correct recognition of the positive probe with respect to the false recognition of the lure probe. This contrast represented the high level of the cognitive conflict. One can clearly see that the two regions from the cerebellum have a much faster initial response for the correct recognition and lower cognitive conflict. It is very important to emphasize, that although the time delay from the retrieval event to the button press for the correct response is on the average shorter than for the false response by 91 ms (1314 ms for the false and 1223 ms for the correct response), this time difference is *much smaller* than the temporal shift observed in Fig. 2a (recall that 1 TR=1.8s).

The cortical region shown in Fig. 2a (right) exhibits, on the other hand, a clear delay in the trailing part of the neural response, which seems to indicate longer activity associated with the false response to the lure probe and higher cognitive conflict.

The regions with significant leading delayed response are listed in Table 1a, and the ones with significant trailing delayed response are listed in Table 1b. The precise criteria and definitions of the relevant observables are given in “Methods”.

**Table 1:** (**a**) Regions in the MMP/CA parcellation with significant leading delayed responses between POScorr and LURfalse.

MMP	CA	AAL	size	x	y	z	$\left\langle {\Delta _{leading} t}\right\rangle$
**(a)**							
L_LIPd	Dorsal-Attention-15_R-Cerebellum	CB lobule 8 R	794	28.8	− 46.8	− 47.4	$3.38 \pm 1.06$
	Visual2-15_R-Cerebellum	CB Crus2 R	347	7.1	− 70.8	− 29.7	$2.37 \pm 0.58$
	Visual1-34_R-Cerebellum	CB Vermis R	324	2.7	− 63.0	− 32.5	$1.77 \pm 0.36$
	Cingulo-Opercular-21_R-Cerebellum	CB lobule 6 R	763	28.2	− 53.6	− 24.0	$1.47 \pm 0.27$
	Dorsal-Attention-15_L-Ctx	IPG L	99	− 29.8	− 55.0	45.5	$1.26 \pm 0.32$
	Dorsal-Attention-17_R-Cerebellum	CB lobule 6 R	22	33.3	− 46.5	− 25.5	$1.07 \pm 0.22$
	Somatomotor-13_R-Cerebellum	CB 4,5 lobule R	707	19.6	− 49.3	− 22.1	$1.03 \pm 0.19$
L_SCEF	Cingulo-Opercular-33_L-Ctx	SMA L	203	− 5.9	1.4	54.8	$0.60 \pm 0.18$
L_AVI	Frontoparietal-44_L-Ctx	INS L	126	− 31.5	23.0	− 4.3	$0.50 \pm 0.14$
**(b)**							
R_a32pr	Cingulo-Opercular-28_R-Ctx	MCC R	127	8.7	26.5	30.5	$1.26 \pm 0.18$
L_8BM	Frontoparietal-32_L-Ctx	SFG L	174	− 4.8	27.2	44.5	$1.25 \pm 0.22$
R_8BM	Frontoparietal-06_R-Ctx	SFGmed R	175	5.9	26.3	44.4	$1.14 \pm 0.17$
L_a32pr	Cingulo-Opercular-55_L-Ctx	ACC L	128	− 7.7	28.1	29.6	$1.09 \pm 0.18$
R_FOP5	Cingulo-Opercular-26_R-Ctx	INS R	156	39.1	26.4	4.2	$0.88 \pm 0.13$
R_AVI	Frontoparietal-20_R-Ctx	INS R	150	33.8	23.7	− 4.4	$0.84 \pm 0.13$
L_AVI	Frontoparietal-44_L-Ctx	INS L	126	− 31.5	23.0	− 4.3	$0.82 \pm 0.13$
R_FOP4	Cingulo-Opercular-19_R-Ctx	INS R	156	38.4	15.6	6.6	$0.78 \pm 0.17$
L_FOP5	Cingulo-Opercular-53_L-Ctx	INS L	138	− 35.9	25.4	4.3	$0.76 \pm 0.13$
L_SCEF	Cingulo-Opercular-33_L-Ctx	SMA L	203	− 5.9	1.4	54.8	$0.47 \pm 0.12$

AAL indicates the AAL region where the centre of mass of the MMP/CA region is located. The MNI coordinates *x*, *y*, *z* of the centre of mass of each region are evaluated as an average over the region coordinates for each subject, as the surface-based cortical data is sensitive to the individual cortical folding patterns. *size* is the number of grayordinates for each region, i.e., voxels for subcortical and vertices for the cortical ones. $\left\langle {\Delta _{leading} t}\right\rangle$ is the average leading time delay (see “Methods” for the definition) between POScorr and LURfalse activations, expressed in units of TR. The errors are estimated by bootstrap. *CB* cerebellum, *INS* insula, *IPG* inferior parietal gyrus, *SMA* supplementary motor area, *L* left hemisphere, *R* right hemisphere. (**b**) Regions in the MMP/CA parcellation with significant trailing delayed responses between POScorr and LURfalse. The columns are as in Table (a) apart from $\left\langle {\Delta _{trailing} t}\right\rangle$, which is the average trailing time delay (see “Methods” for the definition) between POScorr and LURfalse activations, expressed in units of TR. The errors are estimated by bootstrap. *ACC* anterior cingulate cortex, *INS* insula, *MCC* middle cingulate cortex, *SFGmed* superior frontal gyrus medial part, *SMA* supplementary motor area, *L* left hemisphere, *R* right hemisphere.

#### Late stage of the neural response for the POScorr–LURfalse contrast

A very surprising phenomenon occurs quite late after the retrieval event. We observed a group of regions with a significantly higher activity for the correct answer than for the false answer around 5–9 TR after the retrieval event.

**Figure 3:**
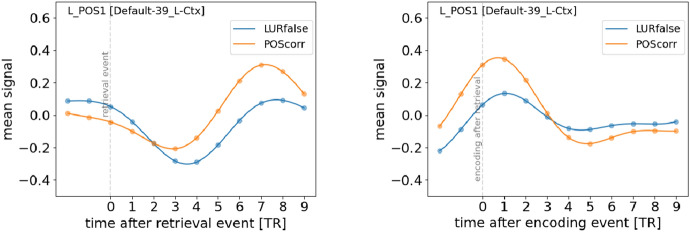
Mean signals for a region exhibiting a significant difference in the neural responses quite late after the retrieval event. On the left, the signals are temporally tied to the retrieval event. On the right, the signals are temporally tied to the encoding event *following* the retrieval event.

To this end, we first selected regions for which the interpolated mean signals of both events have a local maximum in the interval 5–9 TR. The 17 statistically significant regions for the contrast POScorr vs LURfalse (as measured by the area between the mean activities and permutation tests, see “Methods”) are shown in *Supplementary Information* Table A2. The temporal profiles of the mean neural responses for the most relevant region are shown in Fig. 3 (left).

Since the time delay of the neural reaction occurs so late after the retrieval event, one has to be very careful to ensure that the effect is really associated with the retrieval event. Below, we provide arguments that this is indeed so.

First, for the region shown in Fig. 3 (left), we observe a very clear difference in the magnitude w.r.t. correct vs false response. This can be quantitatively seen in two independent ways: (i) the area between the curves is much higher than the critical value from permutation tests (see *Supplementary Information* Table A2) and (ii) the bootstrap error of this area is significantly smaller than its value. Therefore, the neural processing in this region is clearly tied to the correctness of the response given to the retrieval event, even though the processing occurs so late after the event.

Second, the maximum of the neural response occurs roughly 7 TR after retrieval, which is already around the time the participants are shown the next batch of images in the following *encoding* event. One can wonder then whether the observed activity should not be associated to that following *encoding* event. We can answer this question by constructing mean signals for the activity of the same region, but now temporally locked to the time of the *following encoding event*. Note that the time delays between retrieval events and the following encoding events were not fixed but had some random spread. The relevant curves are shown in Fig. 3 (right). We observe that there was already a very substantial rise in the activity *before* the encoding event took place. Indeed, the encoding event is almost at the top of the peak, hence it cannot be considered to be the neural source of the activity.

Let us note that the pattern of behaviours of the regions shown in *Supplementary Information* Table A2 could not be observed using the standard GLM methodology of an HRF tied directly to the retrieval event. The analysis of temporal profiles of mean events proposed in the present paper shows thus its versatility and opens up prospects for observing novel phenomena.

### Early and late stage of neural responses for the NEGcorr–LURcorr contrast

It is instructive to compare the results discussed above with the case when the subject is shown one of two types of *false* images—one which is clearly different from the ones shown in the encoding stage (NEGcorr) or one which is quite similar (LURcorr). In both cases, the subject gives the correct answer, but the difference lies in the level of cognitive conflict. The statistically significant regions for the contrast NEGcorr–LURcorr in the early period (0–5 TR) and late period (5–9 TR) are shown in *Supplementary Information* Tables A3 and A4, respectively.

**Figure 4:**
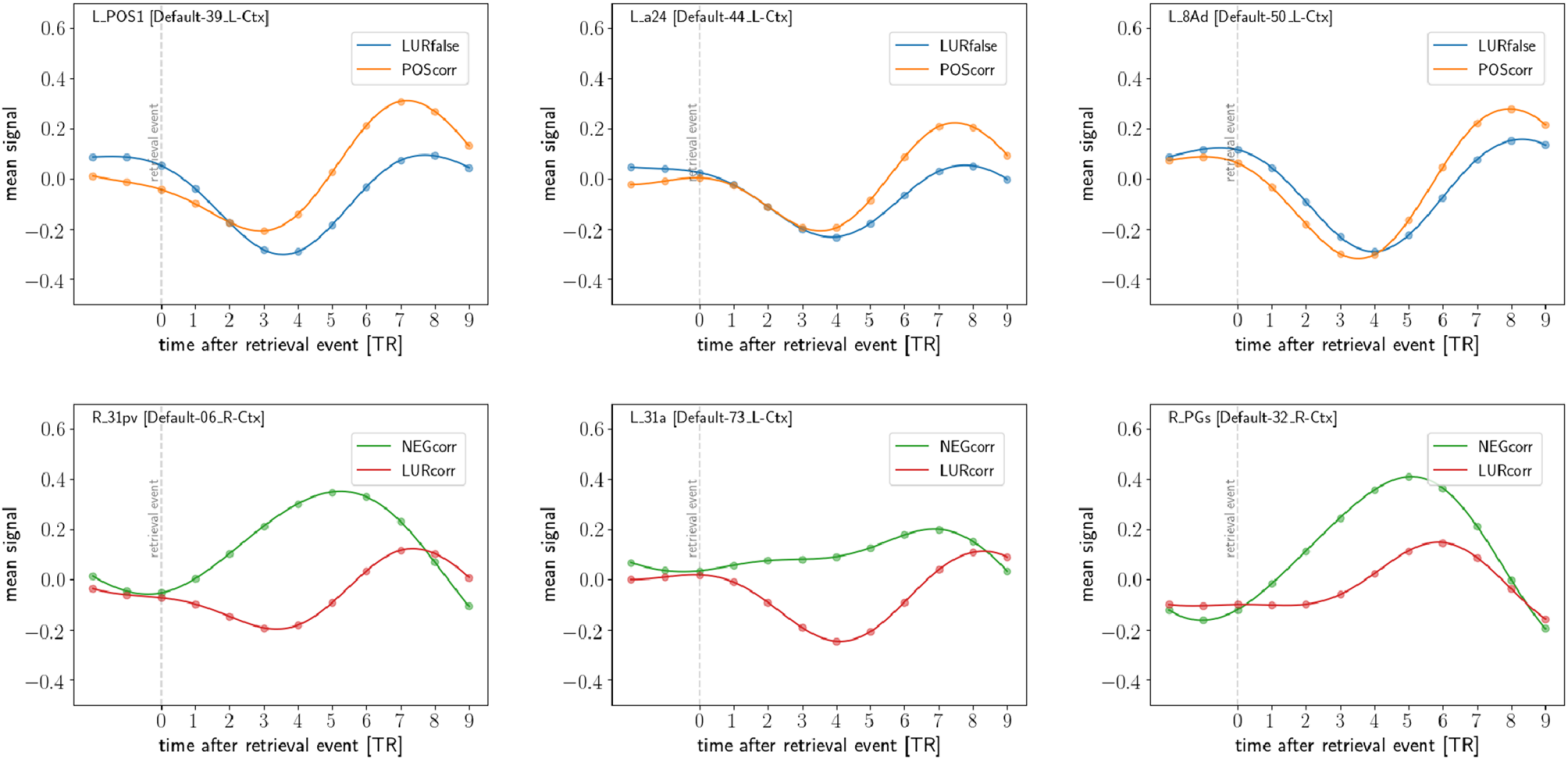
Selected regions with maxima in the period 5–9 TR after POScorr–LURfalse (top) and NEGcorr–LURcorr (bottom) events.

In Fig. 2b we show the mean signals of three selected regions from visual, attentional and executive networks for the contrast NEGcorr–LURcorr, in which the neural response differentiate between two types of stimuli—the higher activity of structures for stimulus with higher cognitive conflict.

For the late stage of neuronal response (5–9 TR), the regions from default mode network were activated (Fig. 4—bottom). It can be easily seen that the course of neural response for stimulus with low cognitive conflict (NEGcorr) looks differently than that for other stimuli. The depicted regions with the greatest difference in the response time-courses are posterior and middle cingulate cortex as well as angular gyrus.

### Comparison of early and late stage for POScorr–LURfalse and NEGcorr–LURcorr contrasts

**Figure 5:**
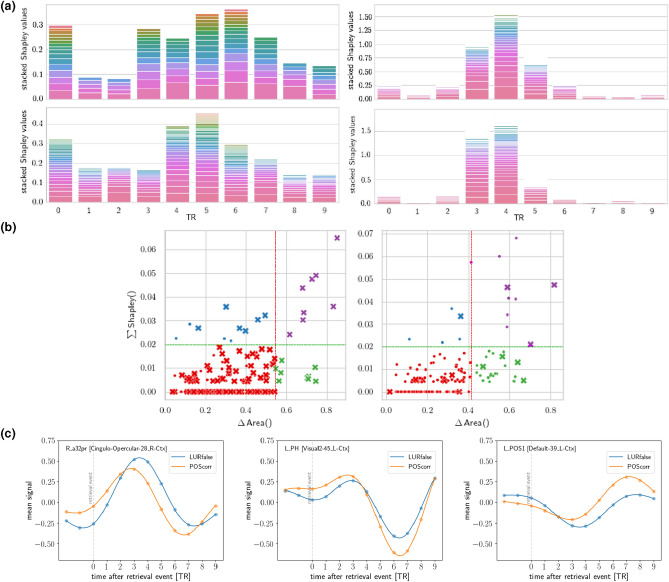
Results of Shapley analysis. (**a**) Stacked histograms show the regions used in the best gradient boosting models, weighted by their Shapley values for each TR on the horizontal axis. For each TR, a stack is composed of bars that correspond to relevant regions. The vertical width of each bar corresponds to that region’s Shapley relative value, and are sorted from the most relevant at the bottom. AAL (top) and MMP/CA parcellations (bottom row) for the POScorr–LURfalse (left) and NEGcorr–LURcorr (right column) problems are given. Histograms show that the same TRs are most relevant irrespective of brain parcellation method and surface/volume registration used. (**b**) The Shapley sum vs $\Delta \,\,Area$ values for POScorr–LURfalse (left) and NEGcorr–LURcorr (right plot) problems and the 0–4 (inclusive) TR time windows. Red vertical and green horizontal lines denote critical values for the $\Delta \,\,Area$ Shapley sums, respectively. Dot and cross marks denote individual regions. Crosses represent regions where local maxima of mean activations for both measures occur. Colors are introduced for readability to denote different critical values quadrants. (**c**) Mean signals temporally locked to the retrieval event for POScorr–LURfalse for region with the highest Shapley sum in the early time window 0–5 TR (left) and regions with the 1st and 4th the highest Shapley sums in the late time window 5–9 TR (centre and right). The relevance of the latter two regions for the ML classifier in the late time window comes from two qualitatively different types of behaviours.

To compare the two contrasts, which are the same in terms of different levels of cognitive conflict, but differ in context of correctness, we depicted the mean signals of the same regions in both contrasts (see *Supplementary Information*, Fig. A5). Regarding the cognitive conflict of varying intensity, we found that for the anterior cingulate cortex (ACC) as well as for the superior frontal gyrus, the course of activity is similar for LURcorr, LURfalse, and POScorr, in contrast to the NEGcorr, when the signal course is flattened and has lower amplitude, respectively. In the angular gyrus, the highest activity is for the less conflicting stimuli (NEGcorr), then for POScorr, and the lowest for both LURcorr and LURfalse. In the case of correctness, the differences in activations of ACC, calcarine gyrus, and superior frontal gyrus were noticed in contrast POScorr–LURfalse, compared to the NEGcorr–LURcorr. The similarity of the signal courses for all depicted regions was noticed in the case of POScorr and LURcorr responses. The contrast with two correct responses (NEGcorr–LURcorr) has lower values of mean signal except for activations from posterior and middle cingulate cortex, and angular gyrus, compared to POScorr–LURfalse contrast. Both the mean signals analysis and Shapley’s analysis showed that in the contrast NEGcorr–LURcorr the information is processed earlier (in TRs 3 and 4) than in the contrast POScorr–LURfalse (TRs 5-7), as shown in Fig. 5a.

### Temporal structure as viewed by a machine learning classifier

An alternative way to assess the relevance of specific regions at particular TRs would be to extract that information from a machine learning model trained to distinguish the given pair of events (like POScorr–LURfalse) based on the parcellated fMRI time series in the 0–9 TR time window following the retrieval event. Concretely, as explained in more detail in the “Methods” section, one can assess the importance of the activation of a given region at a given instant of time for classifying a trial. Aggregating this information over time, we may find the most crucial regions, while aggregating over the regions for a given instant of time will isolate the most informative TR’s for distinguishing the two events. We should note that analysing feature importance and interpretability for nonlinear classifiers is still a very intensely studied topic in the machine learning community^22–24^. In the present paper, we perform a Shapley analysis^24^ (see “Methods” section). Let us contrast this procedure of analysing region importance with the previous analysis using the interpolated mean signals of the activations of individual regions for each event.

In the mean signal analysis, the importance of each region is assessed individually (independently of other regions), and we have full control to impose such conditions as both mean signals having local maxima in given time-windows. On the other hand, the analysis cannot be done for individual trials.

In the machine learning Shapley analysis using a gradient-boosted tree classifier^21^, the regions are analysed in the context of the whole brain (i.e., all other regions and times), even though the Shapley approach aims to isolate as good as it can the contributions of the individual region-time pairs. Moreover, there are no a priori restrictions on the type of activation behaviour used by the algorithm for classifying a given trial. The analysis, however, can be done for individual trials.

Thus, the Shapley analysis might be more difficult to interpret, however, the lack of a priori assumptions may identify patterns in brain activations relevant for distinguishing the events which might have been overlooked in the mean signal analysis. Of course, we would also like to check the consistency of the two very different methods. We leave the possible trial-by-trial analysis for future work.

**Table 2:** The most relevant regions for MMP/CA parcellation POScorr-LURfalse problem with Shapley value *Sh* sums for the regions $R_{TR}$ found to be most important in the 0–4 TR and 5–9 TR time windows.

MMP	CA	AAL	$\displaystyle \sum _{TR\in [0,4]}Sh(R_{TR})$	$\Delta \,\,Area_{TR\in [0,4]}$	$\displaystyle \sum _{TR\in [5,9]}Sh(R_{TR})$	$\Delta \,\,Area_{TR\in [5,9]}$
R_a32pr	Cingulo-Opercular-28_R-Ctx	MCC R	0.0649	0.8525	0.0415	0.5966
	Somatomotor-13_R-CB	CB 4,5 lobule R	0.0491	0.7478		
	Somatomotor-12_R-CB	CB 4,5 lobule R	0.0475	0.7275		
	Dorsal-Attention-18_R-CB	CB lobule 8 R	0.0438	0.6811		
R_AVI	Frontoparietal-20_R-Ctx	INS R	0.0360	0.8335	0.0601	0.5519
R_PGp	Dorsal-Attention-10_R-Ctx	ANG R	0.0358	0.3022		
L_a32pr	Cingulo-Opercular-55_L-Ctx	ACC L	0.0334	0.6867	0.0180	0.5021
L_p10p	Frontoparietal-49_L-Ctx	OFC L	0.0322	0.4954		
L_OP1	Somatomotor-36_L-Ctx	OIFC L	0.0304	0.4593		
L_FOP5	Cingulo-Opercular-53_L-Ctx	INS L	0.0303	0.6829		
R_31a	Frontoparietal-25_R-Ctx	MCC R	0.0285	0.1220	0.0335	0.3635
	Frontoparietal-41_L-HIPP	HIPP L	0.0269	0.1643		
	Auditory-30_R-Thalamus	THA R	0.0268	0.3687		
L_PGp	Dorsal-Attention-22_L-Ctx	ANG L	0.0257	0.3983		
	Frontoparietal-38_R-CB	CB Vermis R	0.0241	0.6168		
L_8Av	Default-49_L-Ctx	SFG L	0.0225	0.0542		
R_Pir	Orbito-Affective-01_R-Ctx	INS R	0.0224	0.2933		
	Visual1-32_R-CB	CB Vermis R	0.0215	0.3263		
L_PH	Visual2-45_L-Ctx	FFG L			0.0682	0.6434
	Default-05_L-CAU	CAU L			0.0574	0.4147
L_POS1	Default-39_L-Ctx	CAL L			0.0474	0.8192
L_a24	Default-44_L-Ctx	ACC L			0.0463	0.5909
R_8BM	Frontoparietal-06_R-Ctx	SFG R			0.0411	0.6318
R_7AL	Somatomotor-07_R-Ctx	SPG R			0.0369	0.3196
	Visual1-24_L-CB	CB Vermis L			0.0341	0.5912
L_AVI	Frontoparietal-44_L-Ctx	INS L			0.0288	0.5893
	Orbito-Affective-03_L-CAU	CAU L			0.0233	0.3581
L_TGv	Language-23_L-Ctx	MTG L			0.0233	0.1134
L_STSda	Language-20_L-Ctx	STG L			0.0219	0.2740
R_POS1	Default-02_R-Ctx	CAL R			0.0210	0.7036

The mean $\Delta \,\,Area_{TR}$ area values are given accordingly for TR regions. *ACC* anterior cingulate cortex, *ANG* angular gyrus, *CAL* calcarine gyrus, *CAU* caudate, *CB* cerebellum, *FFG* fusiform gyrus, *HIPP* hippocampus, *INS* insula, *MCC* middle cingulate gyrus, *MTG* middle temporal gyrus, *OFC* orbitofrontal cortex, *OIFC* opercular part of inferior frontal gyrus, *SFG* superior frontal gyrus, *SPG* superior parietal gyrus, *STG* superior temporal gyrus, *THA* thalamus, *L* left hemisphere, *R* right hemisphere.

Figure 5a shows the Shapley weighted regions aggregated for a given TR for the MMP/CA (bottom row) and AAL (top row) parcellations, which are fairly consistent between each other. We observe, however, a marked difference in the importance of the particular TR for the two pairs of events. In the NEGcorr–LURcorr contrast (right column) the regions in TRs 3 and 4 are essentially sufficient for discriminating the events. For the POScorr–LURfalse contrast (left column), however, we observe also the importance of later moments in time (TRs 5–7) consistent with the earlier analysis using mean signals. The importance of TR 0 is probably due to the fact that the Gradient Boosted Trees classifier does not really use the individual features in isolation, but in the context of others (in particular, if the *difference* of activations between e.g., TR 3 and TR 0 would be significant, then the TR 0 would appear as relevant in the Shapley analysis).

In Fig. 5b we analyse the consistency of the Shapley and the interpolated mean signal analysis. We show a scatter-plot of the aggregated Shapley weights and $\Delta \,\,Area$ for regions in the early and late time windows. We can see that the regions with the highest Shapley weights have also significant $\Delta \,\,Area$ (to the right of the statistically critical value, denoted with a vertical line). In the mean signal analysis, we restricted ourselves to regions with peaks in the relevant time windows (these are marked by crosses in Fig. 5b). In the early time window for POScorr–LURfalse the regions with the highest Shapley indeed have peaks, however in the late time window, this is generically not the case. Indeed, in Fig. 5c (centre) we show the mean time series of the region with the highest Shapley weight in the late period. We observe a significant difference in the depth of the trough of the activation between the two types of events. This kind of behaviour was not taken into account in the mean signal analysis, as there we required that both mean signals have a local *maximum* in the relevant period 5–9 TR. The regions shown on the left and right, however, appear consistently in both methods of analysis.

Tables 2 and A5 (in *Supplementary Information*) show regions that were most relevant in the Shapley analysis for the gradient boosted tree models for POScorr–LURfalse and NEGcorr–LURcorr contrasts and MMP/CA parcellation. The tables show the summary Shapley values for the 0–4 TR and 5–9 TR time windows.

## Discussion

Spatio-temporal patterns of information processing related to erroneous and correct responses were revealed using surface-based fMRI data and machine learning classifiers. It allows us to establish the neural correlates of goal-directed behaviours and cognitive conflict at different levels during a visual short-term memory task. Four types of responses: correct recognition of positive probes (POScorr), correct rejection of lure probes (LURcorr), false recognition of lure probes (LURfalse) and correct rejection of negative probes (NEGcorr) and two types of contrasts (POScorr–LURfalse and NEGcorr–LURcorr) were used.

Neural structures such as prefrontal cortex, insula, anterior cingulate, sensory, and motor cortices detected in presented analysis confirmed the results of a number of previous studies on error commission (for a review, see: REF^25^). Our results of mean signal analysis indicated that the cerebellar regions exhibit a delay for the false response (in the contrast to POScorr–LURfalse) in the ascending part of the neural response (see Table 1a and Fig. 2a). More and more research in recent years has pointed to a role of cerebellum in cognitive functions. It is assumed that the cerebellum has its functional topography, with individual parts responsible for certain functions (motor and non-motor)—as in the cerebral cortex, as well with connections to the regions in the cortex involved in that function^26–28^. The meta-analysis of neuroimaging studies demonstrated the engagement through activations of respective lobules during various cognitive tasks^29^. More specifically, activations of lobules VI, VIIb, VIIIa and Crus were identified in spatial, working memory, and language tasks. Furthermore, the cerebellum is also involved in performance monitoring, error detection, response inhibition, and using error information to improve action execution^30–32^. The recent studies indicated that the cerebellar lobule VIIIa seems to be a part of brain’s visual attention and working memory networks^28^. Our results are consistent with above-mentioned studies, showing cerebellar delay for false recognition of lure probe.

The other regions which show the significantly delayed response for false recognition of the lure probe in the trailing part of the neural response are mostly located in the prefrontal cortex and in the insula (see Table 1b). The prefrontal cortex is involved in cognitive control through storing and manipulating information for actions in the future, and its work is managed by the dopamine neuromodulator. Dopamine influences cognitive control in three distinct ways: gating sensory signals, maintaining stimuli in working memory and sending motor commands^33^. During working memory tasks, the salient stimuli activate dopaminergic neurons in the dorsolateral part of the substantia nigra, which particularly project to the prefrontal cortex, modulating the networks responsible for reacting to environmental changes (among others, executive control)^34^. Dopamine receptors in the prefrontal cortex are linked with the stability of task-goal representations^25^. The delayed response to an error-related stimulus seems to be explained by the modulatory role of the dopamine system, which show long latency responses, up to several seconds^35,36^. The insula, a structure hidden within the lateral sulcus, is involved mainly in sensorimotor and socioemotional processing, however its role was also confirmed in cognitive functions like attention or salience processing^37,38^ as well as in cognitive control together with frontal networks^39^. Singer and colleagues proposed a unifying model assuming the contribution of insula in emotion and uncertainty processing in context of decision-making^40^. Moreover, anterior insula was shown to be involved in performance monitoring and error processing^41^.

Two contrasts of different trial types used in the study allowed us to investigate the effect of cognitive conflict. In the first (POScorr–LURfalse), we compare correct and erroneous responses, in the second (NEGcorr–LURcorr)—two correct responses, but in both, the cognitive conflict at different levels is studied: lower for NEGcorr, higher for POScorr, and the highest for LURcorr and LURfalse. There are both many similarities but also many differences in spatio-temporal patterns of information processing in two studied contrasts. In the case of the contrast POScorr–LURfalse, we found significant activation of the anterior cingulate cortex (ACC), calcarine gyrus, and superior frontal gyrus in comparison to the contrast NEGcorr–LURcorr. ACC plays a major role in conflict processing, error detection, and action selection (for a review, see: REF^42^). The calcarine gyrus located in the primary visual cortex is thought to be responsible for visual information integration and selective attention^43^. Superior frontal gyrus (SFG) as a part of the prefrontal cortex is strongly connected with a variety of brain regions and contributes to many cognitive (especially in working memory) and motor control tasks^44^. Moreover, Hu and colleagues showed that the role of SFG is related to the active control of impulsive responses in a way that activation of the gyrus is correlated with more efficient response inhibition^45^. We found similar signal courses for three types of responses: POScorr, LURcorr, and LURfalse, in the contrast to the NEGcorr response, which has a different signal course in almost all presented regions (see Figs. 2a, b and 4 and *Supplementary Information*, Fig. A5).

When we look at both contrasts from the perspective of correctness, we noticed that the signal courses are similar in the case of POScorr and LURcorr responses. The contrast with two correct responses (NEGcorr–LURcorr) has lower values of mean signal except for activations from posterior and middle cingulate cortex, and angular gyrus. The results related to the level of cognitive conflict showed stronger activation and the course shifted towards the right in the middle cingulate cortex (cingulo-opercular network) for more conflicting stimuli. In the angular gyrus, the highest activity is for the less conflicting stimuli (NEGcorr), then for POScorr, and the lowest for both LURcorr and LURfalse. The previous studies indicated that the angular gyrus is responsible for the conscious prediction of action consequences^46^. For the ACC as well as for the SFG, the course of activity is similar for LURcorr, LURfalse, and POScorr, in contrast to the NEGcorr, when the signal course is flattened and has lower amplitude, respectively. From the functional point of view, the cingulo-opercular network is engaged in maintenance of “tonic alertness” defined as cognitive effortful, self-initiated preparation for information processing and response, conversely to the “phasic alertness”, which is initiated by the stimulus^47^. Posterior cingulate cortex is thought to be responsible for cognitive demands to recall spatial information. Clinical research revealed that lesions of PCC are associated with memory impairments and spatial disorientation^48^.

Shapley’s analysis provided relevant brain regions for two studied contrasts (see Table 2 and *Supplementary Information* Table A5) partly consistent with the analysis of mean signals, which confirms the effectiveness of both methods. The results (see Fig. 5a) allowing to state that information processing related to erroneous response and higher cognitive conflict engages more time and brain areas.

The most interesting result, in our opinion, are the late responses (from 5 to 9 TR) associated with the retrieval (as shown in Fig. 3) and not affected by the encoding process of precise stimuli presented in the next trial. Most of the regions showing differences for correct and false recognitions as well as between more and less conflicting stimuli in late TRs are in the default mode network (DMN), see Fig. 4. Traditionally this network, composed of medial prefrontal cortex, posterior cingulate cortex, precuneus and angular gyrus, is active when the people are not focused on the external tasks, but rather on their inner state or while mind-wandering^49,50^. Its role has been revisited by a recent study of Sormaz and colleagues, showing its activity during ongoing cognition. They also suggested that DMN is active in cognition broadening beyond the off-task state. The results of our study are consistent with the process-memory framework proposed by Hasson et al.^51^, in which timescales of information processing increase along the cortical hierarchy. Using single-unit electrophysiology and fMRI allowed them to discover the timescales of changes in the processing of information on various cortical hierarchy. The longest processing timescales were seen in the areas forming DMN including the angular gyrus, precuneus, posterior cingulate cortex, and medial prefrontal cortex. The results of our study employing the stimuli inducing different levels of cognitive conflict corroborated the mentioned above findings—the stimuli with higher cognitive conflict require longer information processing.

The confirmation of the cortical hierarchy of information processing was possible using single-unit electrophysiology and fMRI, as well as a new methodological approach employed in our study. The use of our methodological approach for EEG data (which have good time resolution) would provide the new insight in cognitive control studies. The previous research revealed that theta oscillations recorded from sensors overlying medial prefrontal cortex, included ACC render efficient cognitive control^52–54^. The theta phase synchronization studies provided an evidence for occurring the integration and exchange of information between brain regions. Furthermore, another EEG study^55^ showed that frontal theta amplitude was significantly higher for unexpected compared to the expected condition. It would be the most interesting to deploy the simultaneous EEG–fMRI to see what is the mechanism of the late responses.

In conclusion, the use of a new methodological approach allows us to determine how the human brain prepares for future events in relation to previous recognition (correct or false) and different levels of cognitive conflict (low and high) in visual short-term memory. The analysis of interpolated mean signals allowed us to uncover a distinct pattern of time delays in the activations of various brain regions. Such an analysis would not be possible with a classical GLM-type investigation. The use of machine learning classifiers, on the one hand, confirmed the observations on the importance of delayed processing and the identification of key relevant regions and on the other hand opened up a possibility of trial by trial studies, which we plan to pursue in the future. This result indicates that machine learning methods are reliable and can be used in the analysis of fMRI data.

To the best of our knowledge, this is the first study showing spatio-temporal patterns of information processing related to erroneous and correct responses aimed at preparing to the adaptive behaviour occurred during retrieval phase, in contrast to previous research which dealt with post-error adjustments related to the encoding process of stimuli presented in the next trial. The brain focusing on the previously-encoded information, provides the evidence that cognition is guided by memory rather than information occurring later in the task. Our results showed that new methods of analysis allow drawing more specific conclusions about neural activity related to cognitive conflict and erroneous and correct responses than the classical methods.

## Methods

### Participants

5354 young and healthy volunteers participated in the first stage of selection via online advertisements on the university website and Facebook. It includes diurnal preference assessment measured by the Chronotype Questionnaire^56^, night sleep quality measured by the Pittsburgh Sleep Quality Index (PSQI)^57^, and daytime sleepiness measured by the Epworth Sleepiness Scale (ESS)^58^. From this step, 451 individuals were selected and identified as morning and evening chronotypes and went through the next stage of selection. The exclusion criteria were sleep problems or excessive daytime sleepiness (as determined by the cut-off points from the PSQI ($\le$ 5 points) and ESS ($\le$ 10 points) questionnaires), drug, alcohol or nicotine dependence, shift work, and travel comprising passing more than two time zones within the past 2 months. The final sample consisted of sixty-five (32 women; mean age: 24.54 $\pm$ 3.43 years old) participants, who completed the selection criteria: age between 20 and 35 years, right-handedness according to the Edinburgh Handedness Inventory (EHI)^59^, normal or corrected-to-normal vision, no neurological or psychiatric disorders, and no MRI contraindications. Informed, written consent was provided by all participants prior to completion of the study procedures. The individuals were remunerated for participation in the experiment. The study was conducted in accordance with the Declaration of Helsinki and approved by the Research Ethics Committee at the Institute of Applied Psychology at the Jagiellonian University.

### Task

The modified short-term memory DRM paradigm^14^ was employed in the study. The task was performed twice—during morning and evening functional magnetic resonance imaging sessions in two versions (A and B). The versions as well as order of sessions were counterbalanced between participants. They were asked to memorize a set consisting of two abstract objects, followed by a mask. Thereafter, a probe was displayed in three conditions: positive (when the probe was in the previously presented set), negative (when the probe was not presented at all) and lure (when the probe was similar on the holistic level to the stimuli in the preceding set). The participants’ goal was to determine whether the stimulus occurred in the previously presented set (right-hand key for “yes”, left-hand key for “no”). The procedure for one trial looks as follows: a fixation point presented for 450 ms, blank screen presented for 100 ms, then the memory set presented for 1800 ms followed by a blank screen (1000 ms) and mask (1200 ms). Afterwards, the probe was displayed for 2000 ms. Duration of the first fixed inter-stimulus interval (ISI) was 1000 ms, the second ISI ranged from 2000 to 16,000 ms (avg. 6097 ms). Mean duration of the inter-trial interval was 8403 ms and ranged from 6000 to 15000 ms. There were 60 memory sets followed by 25 positive, 25 lures and 10 negative probes. The dark gray (RGB 72, 72, 72) stimuli were presented on a light-gray background (RGB 176, 176, 176). The abstract objects (5° wide and 4° high) in memory sets were displayed 3° from the centre of the screen to the left and right, while masks and the objects in memory probes in the centre of the screen. The task was prepared using E-Prime 2.0 (Psychology Software Tools) and presented via a mirror (located on the head coil) on an MR-compatible LCD screen (NordicNeuroLab, Bergen, Norway) with a refresh rate of 60 Hz and a resolution of $800 \times 600$ pixels. The detailed task and procedure description is presented in Ceglarek et al.^60^, however for the convenience of the reader the task procedure is depicted in Fig. A2 (*Supplementary Information*).

### Procedure

One week before the exact study, the duration and quality of sleep were controlled using the MotionWatch8 actigraphs (CamNtech, Cambridge, UK) during the week preceding the study and the experimental days. At the start of the experiment, participants went to the lab to complete a training session (to avoid the influence of the learning process) and to familiarize themselves with the MR lab environment. The training session consisted of three parts. In the first, each participant was informed about the course of the experiment. Next, six experimental trials (2 positive probes, 2 lure probes, and 2 negative ones) were presented to the participant. There was no time limit to familiarize with each trial component, and the participant pressed a key to proceed to the next part of the trial. In the third part of the training session, a whole-task training approach was used. The participants responded to both the probe and distractor by pressing a key with the right or left hand (for “yes” or “no” response, respectively). Stimuli for training differed to those used for the experimental tasks. The participant could complete the task as many times as he/she needed. The possibility of the practice effect was rejected, no differences were found in the performance indices between the first session held for each participant (morning or evening) and the second. The study was conducted on one (when the morning session was the first one) or two (when the morning session was the second one) experimental days. The session order was counterbalanced across participants. They were asked to abstain from alcohol (48 h) and caffeine (24 h) before study and during the experimental days. During days of exact study, they could engage in non-strenuous activities. The night before the morning session, participants slept in rooms located in the same building as the MR laboratory. In the analysis, we look at the response types regardless of the time of day.

### Imaging data acquisition

MRI data were acquired using a 3T Siemens Skyra MR System with a 64-channel coil. For anatomical reference, a T1-weighted MPRAGE sequence was performed (TR = 2.3 s, TE = 2.98 ms, FA = 9°, 176 sagittal slices, slice thickness = 1.1 mm, FOV = 256 × 256 mm). For the BOLD imaging, a T2*-weighted EPI sequence was used (TR = 1.8 s, TE = 27 ms, FA = 75°, 34 slices with interleaved acquisition, voxel size = 4 × 4 × 4 mm, slice thickness = 4 mm, inter-slice gap = 0 mm, FOV = 256 × 256 mm). The 709 volumes were acquired during task performance. Participants’ eye movements were monitored using an eye tracking system (Eyelink 1000, SR research, Mississauga, ON, Canada).

### Volumetric MR data preprocessing

Data preprocessing was performed using the Statistical Parametric Mapping software package (SPM12, Welcome Department of Imaging Neuroscience, UCL, London, UK; www.fil.ion.ucl.ac.uk/spm/) and DPABI (V4.2)^61^ implemented on MATLAB (Mathworks, Inc., MA, USA). Scans were slice-timed corrected and realigned by inclusion of field maps. Following motion correction, each individual’s structural T1-weighted image was co-registered and spatially normalized to Montreal Neurological Institute (MNI) space. The normalized volumes were smoothed using a 4 mm FWHM Gaussian kernel to increase the signal-to-noise ratio of the data. Then the band-pass filtering (0.01-0.08 Hz) was applied. Additionally, the time series for structures from Automated Anatomical Atlas (AAL)^62^ were extracted.

### Surface-based MR data preprocessing

The raw fMRI BIDS data were converted to the HCP style surface-based data using the ciftify^63^ tool (we used the tigrlab/fmriprep_ciftify:v1.3.2-2.3.3 Docker image), which incorporated preprocessing using *fMRIPrep* 1.3.2^64^, as well as parts of the HCP minimal preprocessing pipeline^65^. The description of the anatomical and functional preprocessing steps is adapted from the boilerplate output of fMRIPrep.

- *Anatomical and functional data preprocessing* Two T1-weighted (T1w) images for each subject were corrected for intensity non-uniformity (INU). Brain surfaces were reconstructed using recon-all FreeSurfer 6.0.1^66^, spatial normalization to the ICBM 152 Nonlinear Asymmetrical template version 2009c was performed through nonlinear registration with antsRegistration (ANTs 2.2.0). Brain tissue segmentation of cerebrospinal fluid (CSF), white-matter (WM) and gray-matter (GM) was performed on the brain-extracted T1w using fast FSL 5.0.9^67^.
  For each BOLD run, a reference volume and its skull-stripped version were generated using a custom methodology of *fMRIPrep*. A deformation field to correct for susceptibility distortions was estimated based on a field map that was co-registered to the BOLD reference, using a custom workflow of *fMRIPrep* derived from D. Greve’s epidewarp.fslhttp://www.nmr.mgh.harvard.edu/%7egreve/fbirn/b0/epidewarp.fsl and further improvements of HCP Pipelines^65^. An unwarped BOLD reference was calculated for a more accurate co-registration with the anatomical reference, and then co-registered to the T1w reference. BOLD runs were slice-time corrected using 3dTshift from AFNI 20160207, then were subsequently resampled to MNI152NLin2009cAsym standard space, generating a preprocessed BOLD run in MNI152NLin2009cAsym space. Global signals within the CSF and WM were extracted.
- *Surface-based data preprocessing* The data obtained above were transformed to the composite surface-based cortical and volume based subcortical cifti format by the ciftify tool using MSMSulc surface realignment^68^. The resulting BOLD signal was smoothed using a 4-mm FWHM kernel (taking into account cortical surface distances and boundaries of subcortical structures), detrended, band-pass filtered (0.01–0.1 Hz) and the CSF and WM signals were regressed out.

### Comparison of volumetric and surface based data

As the present data were collected in a conventional manner, not optimized towards the HCP-like pipeline^65^, and the surface-based data were obtained using ciftify^63^—a tool for converting legacy MR acquisitions (see “Methods”), we investigated whether the benefits also apply in our case.

To this end, we take a pair of retrieval events (moments when participants had to recognize or not the previously presented stimuli and give a motor response) and compare the performance of machine learning classifiers in predicting the type of retrieval event (e.g., POScorr vs LURfalse) from the brain activations using either conventional volumetric data or the HCP-style surface-based data. Note that the classifier is trained and evaluated on data coming from multiple participants, hence its performance reflects the inter-subject consistency of the brain activation data.

In Fig. 1a we show the cross-validated performance of a *logistic regression* classifier trained on the grayordinate or conventional voxel based data at a fixed given time frame after the retrieval event. We observe that, indeed, the surface-based data yields consistently better performance across all time frames. We will return to the time-dependence of the performance shortly.

Since the dimensionality of either grayordinates (91,282) or voxels (68,241) is very high, in the analysis of this paper we will mostly use parcellated data. In Fig. 1b we compare the performance of various machine learning classifiers (see “Methods”) trained on parcellated activations in the period 0–9 TR after retrieval event. For the surface-based data we use the Cole-Anticevic parcellation^69^ (CA) which extends the Multi-Modal-Parcellation^70^ (MMP) comprising 360 cortical regions by another 358 subcortical regions. For the volumetric data, we use here the standard Automated Anatomical Atlas (AAL)^62^ parcellation. We observe that all classifiers perform better on the CA parcellated surface-based data than on the volumetric AAL data. Hence, for subsequent analysis, we will employ the former.

### Statistical analyses for behavioural data

Behavioural data analyses were performed using SPSS v27 (IBM Corp., 2020) software. The general linear model (GLM) with accuracy and reaction times as dependent variables and probe types as fixed factor was performed. The probe types were: POScorr (correct recognition of positive probe), POSfalse (erroneous response for positive probe), LURcorr (correct rejection of lure probe), LURfalse (erroneous response for lure probe) and NEGcorr (correct rejection of negative probe). Due to almost 100% correctness for negative probes, the erroneous responses for them were excluded from the analysis. The post-error slowing (PES) was calculated by t tests comparisons of reaction times for all trials and trials after erroneous responses for positive and lure probes, separately. The significance level was set to $p<0.05$ throughout the analysis, Bonferroni corrected, additionally the effect size was computed through partial eta squared ($\eta _{p}^{2}$).

### GLM analysis

The general linear model of SPM12 was used to conduct the fMRI data analyses. At the first level, the event-related design was modelled for each condition during encoding and retrieval phases (correct and false recognitions of positive probe, correct rejections of lure, false recognitions of lure, correct rejections of negative probe) with onsets and durations of presentation of the stimuli, and convolved with a canonical hemodynamic response function. Durations of stimuli during the retrieval phase were elongated to 9 seconds (5 TR) except the trials with the shortest ITI, in which they were elongated to 8 seconds. The eight contrasts for each subject and session were constructed (correct recognition of positive probe $>~$false recognition of lure probe, false recognition of lure probe > correct recognition of positive probe, correct rejection of lures > false recognition of lure probe and false recognition of lure probe > correct rejection of lures at encoding and retrieval). The negative probes were not included in contrasts due to the small number of probes in the task. The contrasts for all participants and two sessions were included into group analysis. The results are presented at cluster-wise $p < 0.05$ level with FDR correction for multiple comparisons and a cluster size of at least 10 voxels.

### Machine learning classifiers

Various machine learning methods were considered in the classification experiments. The first group included linear models: logistic regression, linear Support Vector Machines (linear SVM), and Support Vector Machines with an RBF kernel (rbf SVM). The best results were obtained by a gradient boosting model.

#### Training and validation procedure

Each of the considered is optimized by 5-fold cross-validation, in which the dataset was split into 5 subsets with each used as test data and the rest of the parts are taken as train data. The procedure is repeated 5 times and results averaged. The performance of the selected hyperparameters is measured on a dedicated evaluation set that was not used during the model training step. We calculated the average and standard deviation of the score of AUC for these 5 test datasets and report them in Fig. 1b. We consider the following set of hyperparameters

- regularization parameter *C* from set $\{0.01, 0.1, 1, 10, 100, 1000\}$ for all above methods,
- kernel coefficient *gamma* as a set $\{0.0001, 0.001, 0.01, 0.1, 1\}$ for rbf SVM.

We use a balanced train dataset during the learning models, one batch or train dataset contains the same proportion of active and inactive classes.

#### Logistic regression

Logistic regression^71^ is a technique borrowed by machine learning from the field of statistics. It is similar to linear regression, since it finds an equation that predicts an outcome for one dependent binary variable from one or more independent variables. However, unlike linear regression, the independent variables can be categorical or continuous. To predict class membership, logistic regression uses the log odds ratio rather than probabilities and an iterative maximum likelihood method rather than the least squares to fit the final model. Logistic regression is relatively fast compared to other supervised classification techniques such as kernel SVM but suffers to some degree in its accuracy. For the CA data, the mean of AUC for 5-fold cross-validation equals 0.619 and 0.748 for the POScorr–LURfalse and NEGcorr–LURcorr problems, respectively. For the AAL data, the mean of AUC is equal to 0.587 and 0.624.

We also considered a more accurate Support Vector Machine^72^, a supervised machine learning algorithm that can be used for both classification or regression challenges. SVM constructs a hyperplane or set of hyperplanes in a high- or infinite-dimensional space to separate classes. For this purpose, SVM chooses the extreme points that help in creating such a hyperplane, the support vectors. For nonlinear separation problems, a “kernel trick” is used to transform the input data space into a higher (even infinite) dimensional space and search there for the best dividing hyperplane. SVM offers very high accuracy compared to other classifiers such as logistic regression, together with theoretical assurances on generalization. The mean of AUC for 5-fold cross-validation for CA data are 0.652, 0.719 for the POScorr–LURfalse and NEGcorr–LURcorr problems, respectively. Similarly, for AAL data we get 0.622, 0.669. A detailed comparison of the results for each model can be found in the Fig. 1b.

#### Neural networks

Artificial Neural Networks (ANN) are widely used today in many applications, in particular classification oriented. For ANN classifiers we use here a multilayer perceptron (MLP) classifier with a one, two, or three hidden layers with ReLU nonlinear activation function $ReLU(x) = \max (0, x)$, where *x* is the weighted input to a neuron. At the end of a neural network, we apply a standard logistic function (*sigmoid*) defined as $f(x) = 1/(1+e^{-x})\in [0, 1]$. Similarly to previous methods, we use a 5-fold cross-validation during learning MLP where at each time step of training, the partial derivatives of the loss function with respect to the model parameters are computed to update the weight parameters. As a loss function, we take the binary cross-entropy loss function which is computed for the target and the output discrepancy of MLP, i.e.,

1 $$\begin{aligned} \mathcal {L}\big ((x_i)_{i=1\dots {}N}, (y_i)_{i=1\dots {}N}\big ) = \frac{1}{N}\sum _{i=1}^N y_i\cdot \log (x_i) + (1 - y_i)\cdot \log (1 - x_i), \end{aligned}$$

where $y_i\in \{0, 1\}$ is target and $x_i$ is the predicted probability by model (output value of sigmoid). Learning occurs in the perceptron by iteratively adapting connection weights after each piece of data is processed, based on the error value defined as the difference between the output computed and the expected result. This is an example of supervised learning is carried out with back propagation. For this model, we obtained the mean AUC for CA data of 0.667 and 0.837 for POScorr–LURfalse and NEGcorr–LURcorr problems, respectively. Similarly, for AAL data we obtain 0.634, 0.785. Compared to the previous classification methods, this model gives comparatively better results, see Fig. 1b.

#### Gradient boosting

Gradient boosting is an ensemble model which constructs new classifiers, i.e., ensemble elements, by performing gradient descent in a functional space of models trained^73^. An ensemble is built of several models, usually homogeneous, where each is used to predict the outcome. All the predictions are merged together, either by taking a mean or by voting (usually weighted), to compute the final outcome. This approach reduces the generalization error of the whole model, by driving down the variance term^74^.

The construction of ensembles follows first a weak model hypothesis which states that a group of models performing just a bit better than random, i.e., are *weak* models, gives a strong model provided that the weak models are diverse^75^. Models are said to be diverse if they commit errors in different areas of the data space. Second, the gradient boosting approach, in a process of *additive learning*, constructs a series of models $f_k()$, such that $f_{k+1}(x)=f_k(x)+h_k(x)$. The new added hypothesis $h_k(x)=y-f_k(x)$, where *y* is the true value, is equal to the gradient of the cost function (a squared error cost function in this formulation). The new hypothesis $h_k()$ is found using gradient descent in the space of models.

In the experiments, we used a CatBoost model^21^, which builds a forest of decision trees. We applied a grid search to find the optimal parameters, and then applied a 5-fold cross validation procedure. After finding optimum parameters, we applied Shapley analysis (see “Methods”) to reduce the regions and TRs used; thus the *untuned* and *tuned* models in Fig. 1b.

For MMP/CA parcellation POScorr–LURfalse the mean AUC for untuned model was 0.654 (AAL: 0.631) while for a tuned one for CA 0.776 (AAL: 0.693). The mean AUCs for NEGcorr–LURfalse MMP/CA parcellation untuned model was 0.837 (AAL: 0.790), while for tuned CA model it was 0.882 (AAL: 0.806). The tuned models used about 15% of features.

### The analysis of mean signals

The analysis uses surface-based HCP-style data^19^ which were obtained using the ciftify tool^63^ from the original raw data, as described earlier in the “Methods” section. At each TR, the data contains 91282 *grayordinates* comprising 59412 cortical vertices and 31870 subcortical voxels representing neural subcortical structures.

In order to uniformize the data across participants and sessions, for each session, the time signal of each grayordinate was demeaned and normalized to have unit standard deviation. The resulting signals were parcellated using the Cole–Anticevic methodology^69^ (CA), which is an extension of the cortical Multi-Modal Parcellation^70^ (MMP) to the subcortical structures. In order to better compare regions of different size, the parcellated signal for each region was divided by its standard deviation across concatenated participants and sessions.

For each type of event (POScorr, LURfalse, NEGcorr, LURcorr), 12 temporal frames were extracted—2 preceding and 10 following each retrieval event. We collected in total 2159 POScorr, 903 LURfalse trials, 1060 NEGcorr and 1747 LURcorr trials. These signals were then averaged across all trials of a given type, producing the mean signals for the appropriate events. Finally, the mean activations were interpolated using splines giving the final signals which were used for all subsequent analysis.

The interpolation allows localizing the positions of local maxima and minima of the mean signal, which take into account the global temporal dependence of the activations. This is a much finer measure than the positions of local minima and maxima of the original signal at integer multiplies of TR (see Figs. 2a and 3 for a variety of examples).

#### Observables

We quantify to what extent the mean signals of the same region are different between two events *A* and *B* by measuring

2 $$\begin{aligned} \Delta \,\,Area= \frac{1}{T_f-T_i} \int _{T_i}^{T_f} |y_A(t)-y_B(t)| dt, \end{aligned}$$

where $y_A,\,y_B$ are the interpolated mean signals for the respective events. For the early stage of the signal we take $T_i=0$ and $T_f=5$, while for the late stage analysis we use $T_i=5$ and $T_f=9$ (time measured in TR after the retrieval event). In order to study temporal delays in the neuronal reactions, we can, e.g., compare the peaks (defined through a maximum in an appropriate time period) associated to the two events.

Determining a delay in the rising or trailing parts of the signal (of the type shown in Fig. 2a) is more subtle. Let us consider for definiteness the rising part of the signal. We first form straight lines joining the preceding minimum $(t_0, y_0)$ and the maximum $(t_1,y_1)$ for each of the events, and parametrize them by linear functions $t_A^{lin},\, t_B^{lin}$. We denote the common part of their domain of definition by $[y_-,y_+]$:

3 $$\begin{aligned} y_- = max(y_0^A, y_0^B) \quad \quad \quad \quad y_+ = min(y_1^A, y_1^B). \end{aligned}$$

We can now define the average leading time delay as

4 $$\begin{aligned} \left\langle {\Delta _{leading} t}\right\rangle = \frac{1}{y_+ - y_-} \int _{y_-}^{y_+} \left( t_B^{lin}(y) - t_A^{lin}(y) \right) dy. \end{aligned}$$

Note that the mean activations shown in Fig. 2a (left, centre) rise almost exactly in *parallel*. In order to quantify this behaviour, we define in addition

5 $$\begin{aligned} \left\langle {\Delta _{leading}^2 t}\right\rangle = \frac{1}{y_+ - y_-} \int _{y_-}^{y_+} \left( t_B^{lin}(y) - t_A^{lin}(y), \right) ^2 dy \end{aligned}$$

and measure

6 $$\begin{aligned} \sigma _{leading} = \sqrt{ \left\langle {\Delta _{leading}^2 t}\right\rangle - \left\langle {\Delta _{leading} t}\right\rangle ^2 }. \end{aligned}$$

Small $\sigma _{leading}$ should now pick out the parallel rise in Fig. 2a (left, centre). For the precise criterion, see the following subsection. Finally, in order to avoid unreliable results, we will restrict the computations to regions for which the common domain $[y_-,y_+]$ is large enough. This can be quantified using the ratio

7 $$\begin{aligned} range_{leading} = \frac{y_+ - y_-}{max(y_1^A, y_1^B) - min(y_0^A, y_0^B)}. \end{aligned}$$

We define the corresponding observable for the delay in the trailing part of the signal in an analogous way.

#### Permutation testing, bootstrap and criteria for selecting regions

In order to reliably select the relevant regions out of the 718 regions in the Cole–Anticevic parcellation and avoid the Multiple Comparison problem, we perform permutation testing. To this end, we collect together the trials corresponding to the pair of events under investigation (like POScorr–LURfalse), permute the event labels and compute $\Delta \,\,Area$ for each region, using the permuted labels. We repeat the procedure 1000 times, generating our null distribution. Each time, we compute the *maximum* value of $\Delta \,\,Area$ across all regions. We then determine its 0.95 quantile as the critical value $\Delta \,\,Area(critical)$. The relevant regions are then selected by the criterion $\Delta \,\,Area>\Delta \,\,Area(critical)$. For the pair POScorr–LURfalse in the early stage (0–5 TR) we find $\Delta \,\,Area(critical)=0.5453$ (which selects 31 regions (we neglect two regions with less than 10 grayordinates), see *Supplementary Information* Table A1), while for the late stage (5–9 TR) we obtain $\Delta \,\,Area(critical)=0.4138$ (which selects 17 regions, *Supplementary Information* Table A2). For the pair NEGcorr–LURcorr in the early stage (0–5 TR) we find $\Delta \,\,Area(critical)=0.5280$ (which selects 87 regions, *Supplementary Information* Table A3), while for the late stage (5–9 TR) we obtain $\Delta \,\,Area(critical)=0.4162$ (which selects 14 regions, *Supplementary Information* Table A4).

As the observables that we define for measuring time delays are rather non-trivial, the only way to assess their statistical error is by performing a bootstrap procedure. Again we collect together the *N* trials corresponding to the two events of interest, and construct 1000 bootstrap datasets, each time sampling with replacement *N* trials out of the initial collection, preserving the event labels of the trials. For a given observable, we evaluate it in each of the 1000 bootstrap datasets and take the standard deviation as an estimate of statistical error. We take the ratio of the mean to the standard deviation to define *z*(*observable*).

In order to select the regions listed in Table 1a, with a clear time delay between the rising parts of the neural activations, as seen in Fig. 2a (left, centre) we adopt the following criteria:

1. The region has to be relevant: $\Delta \,\,Area>\Delta \,\,Area(critical)$
2. The leading time delay is statistically significant (as evaluated by bootstrap): $z\left( \left\langle {\Delta _{leading} t}\right\rangle \right) > 2$
3. The rise of the response to the two events is approximately parallel: $\sigma _{leading} < 0.15$
4. There is at least a minimal overlap for a reliable computation of the leading time delay: $range_{leading} > 0.1$

The same criteria are used to select the regions in Table 1a but with the substitution $leading \rightarrow trailing$. An example of the latter case is shown in Fig. 2a (right).

### Shapley analysis

SHapley Additive exPlanations (SHAP)^24^ is a framework for interpreting given model predictions as a sum of the impact of individual features used, that is based on the Shapley values.

The objective is to explain how, for a given example, each input feature contributes to the difference between mean output and the output for this example, i.e., how decisive is that feature. Thus, a Shapley value^76^ is the average marginal contribution of a feature across all possible subsets of features. The direct method of computing a value for some feature *X* given a model *M* would be to first evaluate the target function for a subset including *X*, then replace *X* with a random value to find the difference—the contribution $\phi _X$ of *X*. This needs to be repeated for all possible feature subsets, where the Shapley value for *X* would be the average *X*’s contribution over all coalitions.

In the case of linear models

8 $$\begin{aligned} \hat{f}(x)=\theta _0+\theta _1x_1+\theta _2x_2+\dots +\theta _nx_M, \end{aligned}$$

for an *M* features model. Then the contribution $\phi _j$ of *j*-th feature $x_j$ is the difference

9 $$\begin{aligned} \phi _j(\hat{f})=\theta _jx_j-E(\theta _jX_j)=\theta _jx_j-\theta _jE(X_j), \end{aligned}$$

that is the difference between $x_j$ impact and the mean impact of the *j*-th feature. The sum of contributions of all features would give the difference between the output for a given example and the mean output

10 $$\begin{aligned} \sum _j\phi _j(\hat{f})=\hat{f}(x)-E(\hat{f}(X)), \end{aligned}$$

which is the *efficiency* property. The $\phi _j$ values can be negative. The other properties supported by a Shapley system are: *symmetry*—if features $x_j$ and $x_k$ contribute identically to $\hat{f}(x)$, then $\phi _j=\phi _k$; *dumminess*—if $\hat{f}(S,x_j)=\hat{f}(S)$ for all subsets *S*, then the Shapley value $\phi _j=0$; and *additivity*—that it is possible to compute the Shapley value for a number of models, e.g., trees, individually and then get their average as the Shapley value^77^.

The computation time grows exponentially with the number of features. A Monte–Carlo approach^23^ would be to approximate repeating *N* times

11 $$\begin{aligned} \hat{\phi }_j=\frac{1}{N}\sum _{k=1}^N\left( \hat{f}(x_{+j}^k)-\hat{f}(x_{-j}^k)\right) , \end{aligned}$$

where $\hat{f}(x_{+j}^k)$ is a prediction for *x* with all values, except $x_j$, sampled from a random *z*, whereas $\hat{f}(x_{-j}^k)$ has $x_j$ taken from *z*. Unfortunately, there is no rule for choosing an appropriate repeat number *N*. The Shapley approach is model-agnostic, i.e., the $\phi$ values can be computed for models not necessarily linear.

In this paper, we have used the SHAP framework^24^ to achieve a twofold advantage. First, to tune the gradient boosting model by removing features found to be less informative. Second, to use the Shapley values of the final gradient boosting model to explain their impact on the main cognition question of the paper. The SHAP model defines an explanation as a linearized explanation model *g* computed on, a so called, simplified features $z'\in \{0,1\}^M$

12 $$\begin{aligned} g(v')=\sum _{j=1}^M\phi _j v_j', \end{aligned}$$

where *M* is the maximum coalition (subset) size, and $\phi _j$ is the appropriate Shapley value. The $v_j'\in \{0,1\}$ corresponds to feature presence in the current coalition; thus an input example *x* corresponds to a coalition with all 1’s.

The possible SHAP infrastructure is a TreeSHAP methodology for tree based models like the CatBoost^21^ gradient boosting used here. Because of the tree structure of individual models in CatBoost, the TreeSHAP makes polynomial time computation of Shapley values possible. For further details of SHAP, we direct the readers to the Lundberg *et al.*’s original paper^24^.

By computing once the Shapley values $\phi _j$ for all available features, we were able to reduce the set of features used by removing these which had the lowest absolute value expected $E(|\phi _j|)$ and retrain the models. It can easily be seen in *Supplementary Information* Fig. A4 that reducing the set of features to about 10% of the original number of features resulted in most accurate models.

Due to the high dimensionality of the parcellated fMRI time series (e.g., $718\times 10=7180$ features for 10 time steps with the MMP/CA parcellation) in comparison to the number of trials (3062 for the POScorr–LURfalse events), we have retained around 15% of the overall number of features (see *Supplementary Materials* for details). Then, for subsequent analysis we used the gradient boosted tree model trained on the reduced feature-space, which achieves greater accuracy (see tuned-GradientBoosting in Fig. 1b). In Fig. 5 we present the results of the Shapley analysis of this model.

The Shapley values of the features used in the final models for each combination of problem / brain region coding make it possible to select the most important regions as utilized by the trained gradient boosting model. We have selected those with the highest Shapley sum values for the early and late regions. Results are given in Table 2, and *Supplementary Information* Tab. A5. The relation of the Shapley and $\Delta \,\,Area$ values for POScorr–LURfalse problem is shown in Fig. 5b.

### Limitations of the proposed methods

The key limitation for the analysis of mean signals is the need to collect a sufficiently large set of data so that the differences of the mean signals associated with different events would be statistically relevant. Since in order to check the statistical relevance we adopt permutation testing, and for estimating the errors we adopt bootstrap method, we do not need to assume any kind of normal distribution of the data.

One should also note that the mean signals are computed for individual regions, hence they are easy to interpret, but may therefore miss information about the overall context of other regions. The complementary Shapley analysis focuses on each region but in the context of all others. Hence, in principle it may be more powerful, but at the cost of being much more difficult to interpret from the neurocognitive perspective.

The Shapley analysis using decision tree forests as base models may sometimes be limited by the models’ susceptibility to building the most simple one that explains the data at hand. This may be coped with by obtaining a large enough number of examples, which is a limitation. On the other hand, the presented methodology for reducing the number of input features used is a tool for counterbalancing it (see Supplementary Materials).

## Supplementary Information

Supplementary Information.
