# Supplementary material for: Neural spatio-temporal patterns of information processing related to cognitive conflict and correct or false recognitions
Source: Sci Rep. 2022 Mar 28;12:5271. doi: 10.1038/s41598-022-09141-9 (PMC8960838; doi:10.1038/s41598-022-09141-9)
Supplement: Supplementary file 1 — Supplementary Information. [file 41598_2022_9141_MOESM1_ESM.pdf]

A Supplementary information

A.1 BOLD activities map

Fig. A.1 shows BOLD maps from GLM analysis for representing different brain activities for POScorr > LURfalse (a) and LURfalse > POScorr (b) contrasts.

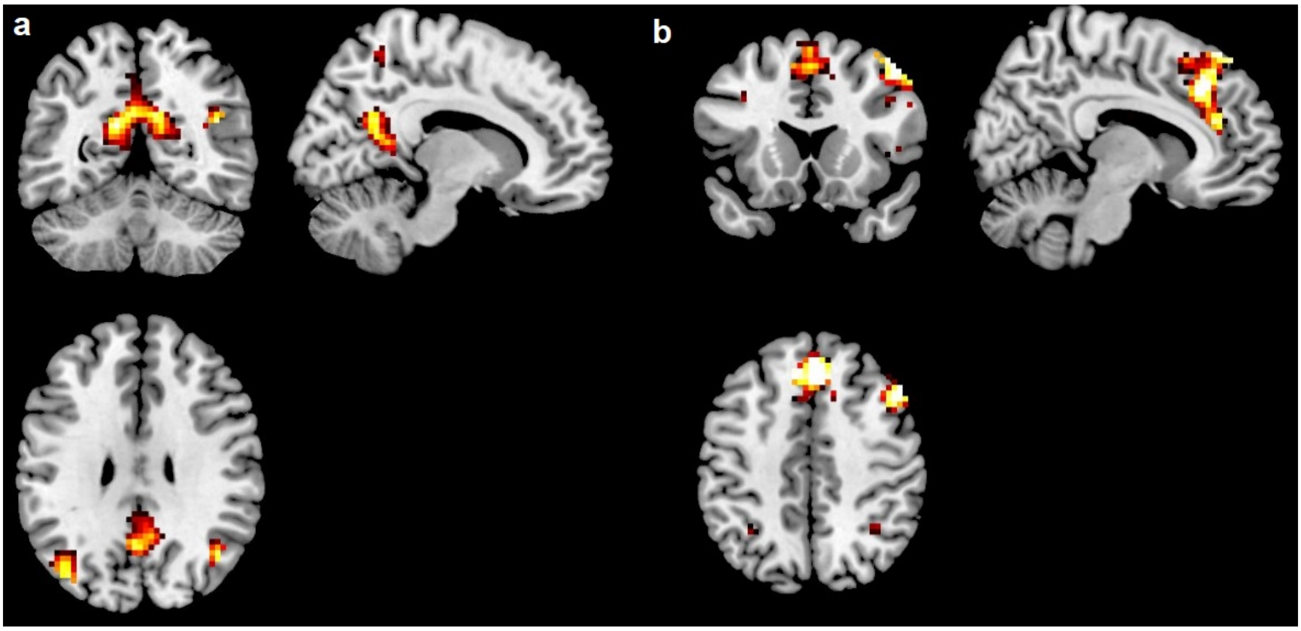

Figure A.1. BOLD activity on the contrasts: a – correct recognition of positive probe versus false recognition of lure probe, b – false recognition of lure probe versus correct recognition of positive probe

A.2 Task procedure

Fig. A.2 shows the example trial of the task presented to the participants.

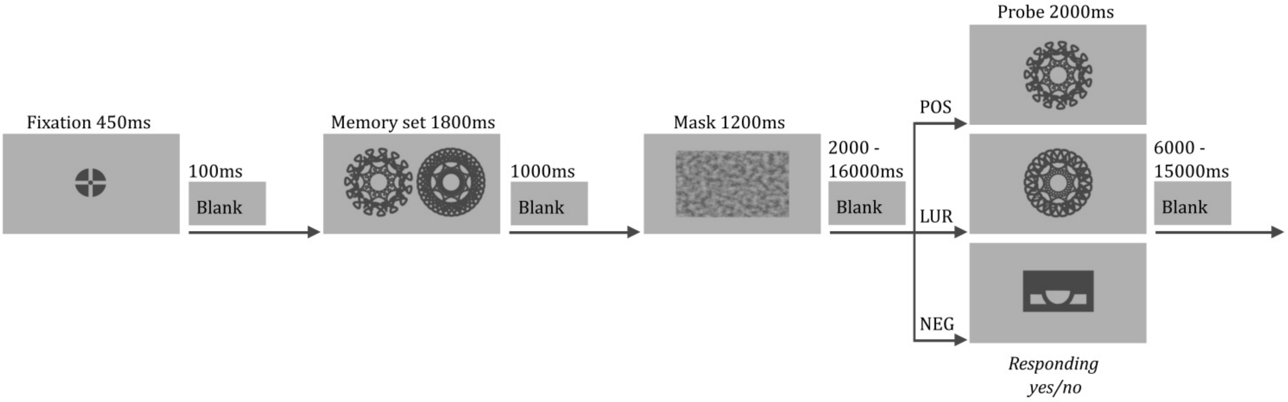

Figure A.2. The sequence of stimuli in the task with an example set of abstract stimuli, POS – positive probe, LUR – lure probe, NEG – negative probe.

A.3 Accuracy statistics

Fig. A.3 give the accuracy statistics, together with reaction times, of all the response types considered in the paper.

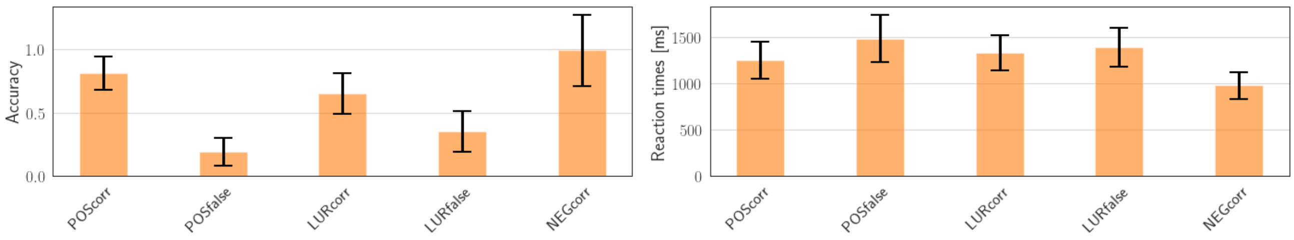

Figure A.3. The accuracy statistics (left side) and reaction times (right side) of all the response types which we considered in our analysis.

A.4 Gradient boosting training

The gradient boosting training was performed using the Catboost implementation[21]. Fig. A.4 shows the Area Under Curve (AUC) values (vertical axis; the higher, the better) from 5-fold cross validations. The values are for AAL (top) and MMP/CA (bottom row) data parcellations of POScorr–LURfalse (left) and NEGcor–LURcorr (right column) experiments. Several models were found by reducing the number of features used by using the Shapley importance values. These values were computed only once at the beginning, therefore the following feature selection was fair. With all features available, the gradient boosting approach (see *Methods* for more details) gives results comparable to other approaches (compare the *untuned Gradient Boosting* bar in Fig. 1 in the main part).

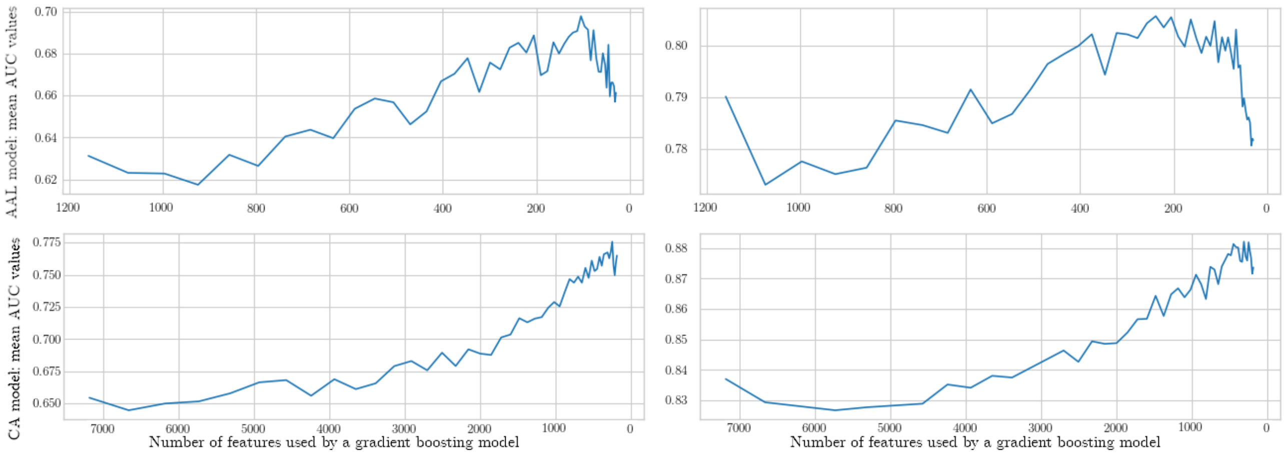

Figure A.4. Mean AUC values (vertical axis) from 5-fold cross validations for the AAL (top) and MMP/CA (bottom row) parcellations with gradient boosting models that use different number of features. Results are shown for the POScorr–LURfalse (left) and NEGcorr–LURcorr (right column). Best models are obtained with 100–200 features for the AAL map, and around 300 features for the MMP/CA parcellation.

On the other hand, the use of all features available might be misleading for classification and lead to *overfitting* when a model starts to fit non-important features. Use of Shapley methodology makes it possible to select features that are the most informative. It suffices to build a model using all the features available (represented with leftmost points on the graphs in Fig. A.4) and compute their Shapley values once. Then, it is possible to select only the features with the highest absolute values to build new models. It can be seen that the best models with the highest mean AUC values use only 10–20% of all the features. For the AAL parcellation the best models use 100–200 features (out of $10 \times 116 = 1160$ total), while for the MMP/CA parcellation it is about 300 (out of $10 \times 718 = 7180$).

A.5 POScorr–LURfalse relevant regions

Tables A.1 and A.2 show the early 0–5 TR and late 5–9 TR window $\Delta\,Area$ relevant regions for the POScorr–LURfalse experiment. To compare with Shapley/GradientBoosting selected features, see Table 2. The critical values for the $\Delta\,Area$ are computed with bootstrap. In Table 2 regions with the highest Shapley sums for the early and late time windows were selected. It can easily be seen that the most important regions using each approach match together. It is important to remember that in case of $\Delta\,Area$ in temporal analysis approach an additional condition was for the region to have a maximum in the time

window, while the ML uses the feature values although separately in each TR, but in the context of other TRs (see Fig. 5 and the discussion of the early and late stage of neural responses in Results section in the main of the paper). The maxima requirement is also why in Table A.2 only Default regions are found, while in Table 2, part for the late window, several others are found.

Table A.1. Regions in the MMP/CA parcellation which are statistically relevant in the early period (0–5 TR) after the POScorr and LURfalse retrieval events and the mean activations for both events have local maxima in the range 1–5 TR. $\Delta\,Area$ is the area between average activations in the range 0–5 TR. The threshold of statistical significance from permutation tests is 0.5453. The errors are estimated by bootstrap. Abbreviations: ACC – anterior cingulate cortex, CB – cerebellum, IFG – inferior frontal gyrus, INS – insula, IOG – inferior occipital gyrus, IPG – inferior parietal gyrus, MOG – middle occipital gyrus, SFG – superior frontal gyrus, SMA – supplementary motor area, STG – superior temporal gyrus, L – left hemisphere, R – right hemisphere.

MMP	CA	AAL	size	x	y	z	$\Delta\,Area$
R_a32pr	Cingulo-Opercular-28_R-Ctx	ACC R	127	8.7	26.5	30.5	0.85 ± 0.10
R_AVI	Frontoparietal-20_R-Ctx	INS R	150	33.8	23.7	-4.4	0.83 ± 0.10
R_FOP5	Cingulo-Opercular-26_R-Ctx	INS R	156	39.1	26.4	4.2	0.77 ± 0.10
	Somatomotor-13_R-Cerebellum	CB lobule 4,5 R	707	19.6	-49.3	-22.1	0.75 ± 0.14
L_AVI	Frontoparietal-44_L-Ctx	INS L	126	-31.5	23.0	-4.3	0.75 ± 0.10
	Cingulo-Opercular-21_R-Cerebellum	CB lobule 6 R	763	28.2	-53.6	-24.0	0.74 ± 0.13
	Somatomotor-12_R-Cerebellum	CB lobule 4,5 R	21	3.3	-67.5	-39.6	0.73 ± 0.14
R_8BM	Frontoparietal-06_R-Ctx	SFG R	175	5.9	26.3	44.4	0.71 ± 0.10
	Visual1-34_R-Cerebellum	CB Vermis R	324	2.7	-63.0	-32.5	0.71 ± 0.14
	Dorsal-Attention-17_R-Cerebellum	CB lobule 6 R	22	33.3	-46.5	-25.5	0.70 ± 0.14
L_a32pr	Cingulo-Opercular-55_L-Ctx	ACC L	128	-7.7	28.1	29.6	0.69 ± 0.10
L_FOP5	Cingulo-Opercular-53_L-Ctx	INS L	138	-35.9	25.4	4.3	0.68 ± 0.10
	Dorsal-Attention-18_R-Cerebellum	CB lobule 6 R	168	20.4	-70.3	-21.8	0.68 ± 0.12
L_8BM	Frontoparietal-32_L-Ctx	SFG L	174	-4.8	27.2	44.5	0.65 ± 0.10
	Dorsal-Attention-12_L-Cerebellum	CB lobule 6 L	181	-18.2	-70.8	-22.4	0.63 ± 0.12
R_LIPd	Dorsal-Attention-03_R-Ctx	IPG R	75	32.0	-53.7	45.8	0.62 ± 0.11
	Visual2-15_R-Cerebellum	CB Crus2 R	347	7.1	-70.8	-29.7	0.60 ± 0.13
R_9-46d	Cingulo-Opercular-15_R-Ctx	IFG L	249	27.6	46.3	23.1	0.59 ± 0.11
R_FOP4	Cingulo-Opercular-19_R-Ctx	INS R	156	38.4	15.6	6.6	0.58 ± 0.10
	Cingulo-Opercular-15_L-Cerebellum	CB lobule 6 R	887	-28.3	-54.8	-24.6	0.58 ± 0.12
	Visual2-10_L-Cerebellum	CB Crus2 R	383	-6.4	-70.9	-31.0	0.58 ± 0.13
	Dorsal-Attention-15_R-Cerebellum	CB lobule 8 R	794	28.8	-46.8	-47.4	0.57 ± 0.15
R_IFJp	Frontoparietal-10_R-Ctx	IFG R	63	40.0	8.0	29.7	0.57 ± 0.11
	Language-13_R-Cerebellum	CB lobule 6 R	14	34.1	-60.6	-24.4	0.57 ± 0.12
L_LIPd	Dorsal-Attention-15_L-Ctx	IPG L	99	-29.8	-55.0	45.5	0.57 ± 0.10
	Somatomotor-11_R-Cerebellum	CB lobule 4,5 R	531	18.3	-57.7	-53.4	0.56 ± 0.14
L_9-46d	Cingulo-Opercular-42_L-Ctx	IFG L	267	-27.7	43.6	24.5	0.56 ± 0.10
L_SCEF	Cingulo-Opercular-33_L-Ctx	SMA L	203	-5.9	1.4	54.8	0.56 ± 0.11
R_PEF	Cingulo-Opercular-02_R-Ctx	STG R	91	45.1	3.1	35.9	0.55 ± 0.10
L_VIP	Visual2-44_L-Ctx	IOG L	116	-20.6	-59.5	56.1	0.55 ± 0.13
L_V1	Visual1-04_L-Ctx	MOG L	831	-10.9	-82.3	1.4	0.55 ± 0.12

A.6 NEGcorr–LURcorr relevant regions

Tables A.3 and A.4 show the $\Delta\,Area$ relevant regions for the 0–5 TR early and 5–9 TR late stages for NEGcorr and LURcorr retrieval events. The given regions have their $\Delta\,Area$ values above appropriate critical values computed using permutation tests (see *Methods*). The additional requirement, to be consistent with the proposed mean signal analysis, is for the regions to have their $\Delta\,Area$ local maxima in the appropriate TR window. The Shapley relevant regions for both early and late regions are given in Table A.5. Because of the maximum requirement for the mean analysis, Table A.4 is composed of only Default regions, while the late stage important regions for the gradient boosting machine learning approach using Shapley approach are composed of other regions too. This is because machine learning approach uses all features, i.e., regions in their TRs, separately, although in context of other features.

Table A.2. Regions in the MMP/CA parcellation which are statistically relevant in the late period (5–9 TR) after the POScorr and LURfalse retrieval events and the mean activations for both events have local maxima in the range 5–9 TR. $\Delta Area$ is the area between average activations in the range 5–9 TR. The threshold of statistical significance from permutation tests is 0.4138. The errors are estimated by bootstrap. Abbreviations: ACC – anterior cingulate cortex, CAL – calcarine gyrus, MCC – middle cingulate gyrus, MFGorb – medial frontal gyrus orbital part, MOG – middle occipital gyrus, PCC – posterior cingulate cortex, PCUN – precuneus, SFG – superior frontal gyrus, L – left hemisphere, R – right hemisphere.

MMP	CA	AAL	size	x	y	z	$\Delta Area$
L_POS1	Default-39_L-Ctx	CAL L	225	-10.6	-58.7	14.3	0.82 ± 0.13
R_POS1	Default-02_R-Ctx	CAL R	212	13.2	-55.9	14.8	0.70 ± 0.13
R_v23ab	Default-04_R-Ctx	PCUN R	82	5.2	-52.6	21.0	0.67 ± 0.13
L_10r	Default-47_L-Ctx	MFGorb L	94	-7.6	51.5	-6.1	0.64 ± 0.13
L_v23ab	Default-41_L-Ctx	PCUN L	72	-4.9	-54.8	21.3	0.62 ± 0.13
L_a24	Default-44_L-Ctx	ACC L	90	-5.4	41.2	-1.6	0.59 ± 0.13
L_PGs	Default-70_L-Ctx	MOG L	305	-39.8	-70.2	35.2	0.57 ± 0.13
R_10r	Default-09_R-Ctx	MFGorb R	74	7.3	50.0	-8.0	0.56 ± 0.12
L_31pv	Default-43_L-Ctx	PCC L	109	-8.7	-47.1	32.3	0.56 ± 0.13
L_7m	Default-38_L-Ctx	SFG L	130	-4.3	-60.3	36.5	0.54 ± 0.13
R_7m	Default-01_R-Ctx	PCUN R	135	5.3	-60.8	35.5	0.52 ± 0.14
L_p32	Default-46_L-Ctx	ACC L	99	-9.2	51.0	1.6	0.50 ± 0.13
L_d23ab	Default-42_L-Ctx	PCC L	88	-3.1	-40.5	33.1	0.49 ± 0.13
L_31a	Default-73_L-Ctx	MCC L	64	-5.2	-39.7	41.6	0.47 ± 0.13
L_8Ad	Default-50_L-Ctx	SFG L	242	-22.4	26.0	43.5	0.47 ± 0.13
L_31pd	Default-72_L-Ctx	PCUN L	108	-9.6	-50.7	37.2	0.45 ± 0.13
R_a24	Default-07_R-Ctx	ACC R	82	5.9	39.5	-1.6	0.44 ± 0.13

A.7 Comparison of complementary regions for POScorr–LURfalse and NEGcorr–LURcorr contrasts

To compare the two contrasts, which are the same in terms of different levels of cognitive conflict, but differ in the context of correctness, we depicted the mean signals of the same regions in both contrasts. Fig. A.5 showed the time courses of brain areas complementary to regions presented in the manuscript.

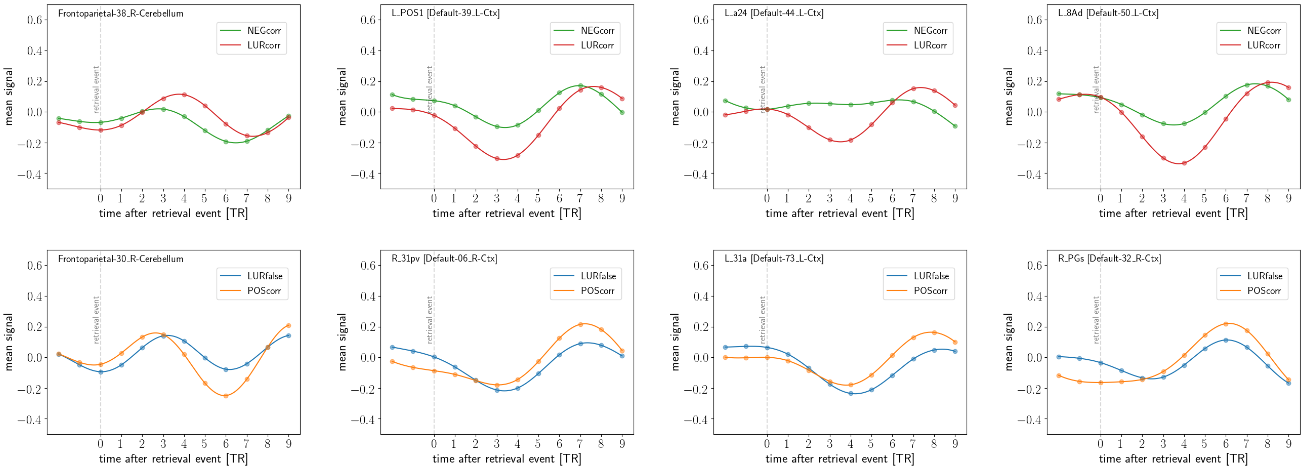

Figure A.5. Time courses of brain regions complementary to regions in Fig. 2a, 2b, and 4 to compare the both contrasts (POScorr-LURfalse and NEGcorr-LURcorr). The first column (on the left) is complementary to regions in Fig. 2a, 2b, and the remaining regions – to Fig. 4.

Table A.3. Regions in the MMP/CA parcellation which are statistically relevant in the early period (0–5 TR) after the NEGcorr and LURcorr retrieval events and the mean activations for both events have local maxima in the range 1–5 TR. $\Delta\,Area$ is the area between average activations in the range 0–5 TR. The threshold of statistical significance from permutation tests is 0.5280. The errors are estimated by bootstrap. Abbreviations: MTG – middle temporal gyrus, INS – insula, MOG – middle occipital gyrus, IPG – inferior parietal gyrus, SFG – superior frontal gyrus, PCC – posterior cingulate cortex, IFG – inferior frontal gyrus, ANG – angular gyrus, MTG – middle temporal gyrus, STG – superior temporal gyrus, IOG – inferior occipital gyrus, ACC – anterior cingulate cortex, MOG – middle occipital gyrus, FFG – fusiform gyrus, SMG – supramarginal gyrus, PCUN – precuneus, CB – cerebellum, THA – thalamus, SMA – supplementary motor area, DCN – diencephalon, CAU – caudate, MFG – middle frontal gyrus, PRE – precentral gyrus, HIPP – hippocampus, BST – brainstem, MCC – middle cingulate gyrus, PCL – paracentral gyrus, PHG – parahippocampal gyrus, L – left hemisphere, R – right hemisphere.

MMP	CA	AAL	size	x	y	z	$\Delta\,Area$
R_STSvp	Default-27_R-Ctx	MTG R	151	57.5	-26.1	-6.0	1.26 ± 0.11
L_AVI	Frontoparietal-44_L-Ctx	INS L	126	-31.5	23.0	-4.3	1.24 ± 0.10
R_LO1	Visual2-12_R-Ctx	MOG R	43	39.9	-79.0	3.8	1.20 ± 0.08
R_AVI	Frontoparietal-20_R-Ctx	INS R	150	33.8	23.7	-4.4	1.17 ± 0.10
R_IP2	Frontoparietal-22_R-Ctx	IPG R	218	42.7	-44.3	43.9	1.17 ± 0.10
R_8BM	Frontoparietal-06_R-Ctx	SFG R	175	5.9	26.3	44.4	1.16 ± 0.11
L_IP2	Frontoparietal-46_L-Ctx	IPG L	198	-38.6	-49.5	41.9	1.15 ± 0.10
L_IP1	Frontoparietal-47_L-Ctx	IPG L	189	-30.9	-65.7	42.3	1.13 ± 0.10
L_8BM	Frontoparietal-32_L-Ctx	SFG L	174	-4.8	27.2	44.5	1.13 ± 0.10
L_RSC	Frontoparietal-29_L-Ctx	PCC L	211	-4.2	-36.0	21.8	1.11 ± 0.14
R_RSC	Frontoparietal-01_R-Ctx	PCC R	231	5.6	-35.5	21.8	1.04 ± 0.14
L_p9-46v	Frontoparietal-37_L-Ctx	IFG L	128	-40.8	27.0	28.9	1.04 ± 0.10
R_IP1	Frontoparietal-23_R-Ctx	ANG R	194	36.7	-62.9	42.1	1.00 ± 0.09
R_TPOJ1	Language-08_R-Ctx	MTG R	297	52.4	-40.0	8.2	1.00 ± 0.12
R_STV	Posterior-Multimodal-02_R-Ctx	STG R	148	55.7	-41.0	18.1	0.99 ± 0.13
L_LO2	Visual2-40_L-Ctx	IOG L	52	-39.5	-82.2	-4.1	0.99 ± 0.10
R_p9-46v	Frontoparietal-12_R-Ctx	IFG R	181	42.5	31.6	26.7	0.99 ± 0.10
L_a32pr	Cingulo-Opercular-55_L-Ctx	ACC L	128	-7.7	28.1	29.6	0.99 ± 0.10
R_LO2	Visual2-13_R-Ctx	IOG R	31	43.1	-78.1	-5.3	0.98 ± 0.10
R_IFSp	Frontoparietal-11_R-Ctx	IFG R	133	44.9	26.1	20.9	0.98 ± 0.11
L_LO1	Visual2-39_L-Ctx	MOG L	37	-36.1	-84.6	5.2	0.96 ± 0.09
R_FFC	Visual2-10_R-Ctx	FFG R	167	39.3	-53.8	-17.4	0.96 ± 0.10
R_STSdp	Language-07_R-Ctx	STG R	210	49.2	-23.9	-4.4	0.96 ± 0.13
R_PFt	Dorsal-Attention-05_R-Ctx	SMG R	193	53.9	-22.8	39.0	0.96 ± 0.10
R_PFm	Frontoparietal-24_R-Ctx	IPG R	471	50.9	-47.4	39.7	0.93 ± 0.12
R_31a	Frontoparietal-25_R-Ctx	PCUN R	72	6.7	-42.1	40.6	0.92 ± 0.14
L_FOP5	Cingulo-Opercular-53_L-Ctx	INS L	138	-35.9	25.4	4.3	0.92 ± 0.10
R_VVC	Visual2-27_R-Ctx	FFG R	124	29.7	-46.8	-17.2	0.91 ± 0.10
	Dorsal-Attention-12_L-Cerebellum	CB 6 lobule L	181	-18.2	-70.8	-22.4	0.91 ± 0.08
R_a32pr	Cingulo-Opercular-28_R-Ctx	ACC R	127	8.7	26.5	30.5	0.89 ± 0.10
L_PFm	Frontoparietal-48_L-Ctx	IPG L	509	-47.2	-53.2	39.3	0.87 ± 0.13
	Frontoparietal-48_R-Thalamus	THA R	449	8.4	-12.3	9.2	0.82 ± 0.10
L_SCEF	Cingulo-Opercular-33_L-Ctx	SMA L	203	-5.9	1.4	54.8	0.82 ± 0.10
L_FFC	Visual2-37_L-Ctx	FFG L	151	-38.4	-55.2	-17.1	0.81 ± 0.09
	Visual1-63_R-Thalamus	THA R	226	13.7	-19.7	7.3	0.81 ± 0.10
R_PIT	Visual2-14_R-Ctx	IOG R	62	38.5	-79.1	-11.9	0.79 ± 0.10
R_6r	Cingulo-Opercular-12_R-Ctx	IFG R	263	50.1	8.8	17.8	0.78 ± 0.09
	Visual1-42_L-Diencephalon	DCN L	307	-12.0	-18.0	-8.2	0.77 ± 0.11
	Posterior-Multimodal-13_L-Cerebellum	CB Crus2 L	57	-18.9	-74.1	-36.4	0.77 ± 0.14
	Frontoparietal-47_L-Thalamus	THA L	470	-7.7	-12.6	8.8	0.77 ± 0.10
	Cingulo-Opercular-11_R-Caudate	CAU R	32	10.3	5.8	7.8	0.76 ± 0.10
R_FOP5	Cingulo-Opercular-26_R-Ctx	INS R	156	39.1	26.4	4.2	0.76 ± 0.09

Continued on next page

Table A.3 – continued from previous page

MMP	CA	AAL	size	x	y	z	$\Delta\,Area$
L_PIT	Visual2-41_L-Ctx	FFG L	62	-37.1	-78.8	-12.4	0.74 ± 0.10
L_VVC	Visual2-54_L-Ctx	FFG L	126	-29.3	-50.6	-17.2	0.74 ± 0.11
	Dorsal-Attention-24_R-Thalamus	THA R	53	15.0	-28.3	7.7	0.73 ± 0.09
L_8C	Frontoparietal-33_L-Ctx	MFG L	257	-40.1	13.1	37.3	0.73 ± 0.10
	Frontoparietal-30_R-Cerebellum	CB Vermis R	58	2.1	-51.6	-33.8	0.73 ± 0.11
	Visual1-59_L-Thalamus	THA L	195	-13.5	-20.4	8.7	0.73 ± 0.10
L_6r	Cingulo-Opercular-40_L-Ctx	PRE L	242	-49.3	5.8	18.4	0.72 ± 0.10
R_PSL	Cingulo-Opercular-03_R-Ctx	STG R	175	60.0	-36.1	23.8	0.71 ± 0.11
	Cingulo-Opercular-38_R-Thalamus	THA R	148	9.7	-15.3	4.4	0.71 ± 0.10
	Cingulo-Opercular-09_L-Caudate	CAU L	32	-9.8	4.2	8.1	0.70 ± 0.10
	Visual1-58_L-Thalamus	THA L	21	-4.2	-23.7	-0.4	0.68 ± 0.10
R_i6-8	Frontoparietal-18_R-Ctx	MFG R	99	32.2	9.7	53.3	0.68 ± 0.10
R_TE1m	Frontoparietal-28_R-Ctx	MTG R	122	60.6	-22.3	-15.5	0.66 ± 0.10
	Visual2-27_R-Hippocampus	HIPP R	103	26.3	-34.6	-1.8	0.66 ± 0.12
	Language-13_R-Cerebellum	CB 6 lobule R	14	34.1	-60.6	-24.4	0.65 ± 0.10
	Cingulo-Opercular-36_L-Thalamus	THA L	120	-8.8	-16.9	4.1	0.65 ± 0.11
	Default-09_R-Caudate	CAU R	15	10.3	22.5	0.3	0.65 ± 0.11
L_POS2	Frontoparietal-30_L-Ctx	PCUN L	250	-9.2	-69.0	36.1	0.65 ± 0.13
	Frontoparietal-16_L-Cerebellum	CB 9 lobule L	36	-4.1	-52.4	-34.3	0.64 ± 0.11
	Dorsal-Attention-23_L-Thalamus	THA L	67	-14.1	-30.1	6.3	0.64 ± 0.10
	Frontoparietal-11_R-Caudate	CAU R	625	13.5	7.8	11.7	0.64 ± 0.11
L_TPOJ2	Posterior-Multimodal-06_L-Ctx	MTG L	156	-48.4	-58.7	10.0	0.64 ± 0.13
L_AAIC	Orbito-Affective-05_L-Ctx	INS L	76	-33.9	11.6	-12.3	0.63 ± 0.13
	Default-08_R-Caudate	CAU R	19	7.2	9.7	-2.7	0.63 ± 0.11
R_IFJa	Language-04_R-Ctx	IFG R	79	42.3	15.9	26.0	0.63 ± 0.10
L_33pr	Cingulo-Opercular-37_L-Ctx	ACC L	55	-2.6	3.8	30.7	0.61 ± 0.10
	Frontoparietal-10_L-Caudate	CAU L	549	-12.7	5.6	12.4	0.61 ± 0.10
	Visual1-57_L-Thalamus	THA L	16	-4.5	-15.9	-4.0	0.61 ± 0.10
	Visual1-10_LR-Brainstem	BST	757	-0.1	-31.5	-22.3	0.60 ± 0.10
R_TPOJ2	Posterior-Multimodal-03_R-Ctx	MTG R	143	53.2	-53.6	7.1	0.60 ± 0.12
	Visual1-34_R-Cerebellum	CB Vermis R	324	2.7	-63.0	-32.5	0.60 ± 0.10
L_PF	Cingulo-Opercular-51_L-Ctx	SMG L	372	-54.6	-37.4	35.1	0.59 ± 0.13
	Visual1-43_R-Diencephalon	DCN R	287	13.7	-18.0	-7.8	0.59 ± 0.10
R_LBelt	Auditory-06_R-Ctx	STG R	85	49.2	-23.7	9.4	0.58 ± 0.13
L_SFL	Language-12_L-Ctx	SMA L	185	-8.1	12.0	59.2	0.58 ± 0.10
L_p47r	Frontoparietal-50_L-Ctx	IFG L	129	-41.1	41.5	3.5	0.57 ± 0.11
L_p24pr	Cingulo-Opercular-36_L-Ctx	MCC L	81	-2.9	-3.8	39.4	0.56 ± 0.14
	Visual1-23_L-Cerebellum	CB 8 lobule L	117	-4.3	-62.1	-38.6	0.55 ± 0.10
R_SCEF	Cingulo-Opercular-06_R-Ctx	SMA R	193	6.7	2.9	55.0	0.55 ± 0.11
L_p32pr	Cingulo-Opercular-39_L-Ctx	MCC L	139	-7.9	12.4	38.9	0.55 ± 0.10
L_6mp	Somatomotor-33_L-Ctx	PCL L	258	-12.9	-13.6	64.6	0.54 ± 0.13
R_33pr	Frontoparietal-04_R-Ctx	ACC R	75	4.1	5.1	30.0	0.54 ± 0.10
	Visual2-24_L-Hippocampus	HIPP L	76	-25.3	-35.8	-3.1	0.54 ± 0.12
	Default-10_L-Cerebellum	CB 9 lobule L	244	-5.6	-53.5	-44.0	0.53 ± 0.13
	Visual1-45_R-Hippocampus	PHG R	61	18.6	-34.6	-3.1	0.53 ± 0.11

Table A.4. Regions in the MMP/CA parcellation which are statistically relevant in the late period (5–9 TR) after the NEGcorr and LURcorr retrieval events and the mean activations for both events have local maxima in the range 5–9 TR. $\Delta\,Area$ is the area between average activations in the range 5–9 TR. The threshold of statistical significance from permutation tests is 0.4162. The errors are estimated by bootstrap. Abbreviations: ANG – angular gyrus, MCC – middle cingulate gyrus, MFG – middle frontal gyrus, MOG – middle occipital gyrus, MTG – middle temporal gyrus, PCC – posterior cingulate cortex, PCUN – precuneus, L – left hemisphere, R – right hemisphere.

MMP	CA	AAL	size	x	y	z	$\Delta\,Area$
R_31pv	Default-06_R-Ctx	PCC R	111	9.1	-46.8	32.5	0.71 ± 0.08
L_31a	Default-73_L-Ctx	MCC L	64	-5.2	-39.7	41.6	0.65 ± 0.10
L_d23ab	Default-42_L-Ctx	PCC L	88	-3.1	-40.5	33.1	0.61 ± 0.07
R_7m	Default-01_R-Ctx	PCUN R	135	5.3	-60.8	35.5	0.55 ± 0.10
L_31pv	Default-43_L-Ctx	PCC L	109	-8.7	-47.1	32.3	0.53 ± 0.07
R_PGs	Default-32_R-Ctx	ANG R	209	45.4	-63.8	35.5	0.53 ± 0.10
L_7m	Default-38_L-Ctx	PCUN L	130	-4.3	-60.3	36.5	0.53 ± 0.09
R_v23ab	Default-04_R-Ctx	PCUN R	82	5.2	-52.6	21.0	0.52 ± 0.07
L_31pd	Default-72_L-Ctx	PCUN L	108	-9.6	-50.7	37.2	0.51 ± 0.08
L_v23ab	Default-41_L-Ctx	PCUN L	72	-4.9	-54.8	21.3	0.50 ± 0.07
L_PGs	Default-70_L-Ctx	MOG R	305	-39.8	-70.2	35.2	0.47 ± 0.07
R_TE1a	Default-29_R-Ctx	MTG R	105	57.6	-4.8	-23.2	0.46 ± 0.10
R_8Ad	Default-12_R-Ctx	MFG R	239	23.4	27.4	43.9	0.45 ± 0.10
R_STSva	Default-37_R-Ctx	MTG R	81	52.8	-7.5	-18.1	0.43 ± 0.09

Table A.5. Most relevant regions for MMP/CA parcellation NEGcorr-LURcorr problem with Shapley value Sh sums given for 0–4 TR and 5–9 TR time windows. Most relevant regions for AAL coded POScorr-LURfalse problem with Shapley value Sh sums for the regions R_{TR} found to be most important in the 0–4 TR and 5–9 TR time windows. Abbreviations: ACC – anterior cingulate cortex, ANG –angular gyrus, CAL - calcarine gyrus, DCN - diencephalon, FFG - fusiform gyrus, HIPP – hippocampus, IFG - inferior frontal gyrus, INS – insula, IOG - inferior occipital gyrus, IPG - inferior parietal gyrus, MOG - middle occipital gyrus, MTG - middle temporal gyrus, PCUN - precuneus, PRE - precentral gyrus, SFG – superior frontal gyrus, SMG - supramarginal gyrus, SPG - superior parietal gyrus, STG - superior temporal gyrus, L – left hemisphere, R – right hemisphere.

MMP	CA	AAL	$\sum_{TR\in[0,4]} Sh(R_{TR})$	$\Delta Area_{TR\in[0,4]}$	$\sum_{TR\in[5,9]} Sh(R_{TR})$	$\Delta Area_{TR\in[5,9]}$
R_IP0	Dorsal-Attention-11_R-Ctx	IPG R	0.2401	0.4620		
R_MIP	Dorsal-Attention-02_R-Ctx	SPG R	0.2124	0.4211		
R_IPS1	Visual2-09_R-Ctx	IOG R	0.2034	0.3971		
R_8BM	Frontoparietal-06_R-Ctx	SFG R	0.1573	0.7106		
R_STSvp	Default-27_R-Ctx	MTG R	0.1485	0.1356		
R_23d	Default-03_R-Ctx	PCUN R	0.1419	0.0433		
L_AVI	Frontoparietal-44_L-Ctx	INS L	0.1358	0.7469		
R_PGi	Default-31_R-Ctx	ANG R	0.1150	0.2341		
L_46	Cingulo-Opercular-41_L-Ctx	PRE L	0.1017	0.2851		
R_LIPd	Dorsal-Attention-03_R-Ctx	IPG R	0.1013	0.6172		
R_V3CD	Visual2-24_R-Ctx	FFG R	0.0812	0.5239		
R_STV	Posterior-Multimodal-02_R-Ctx	STG R	0.0796	0.2054		
L_a32pr	Cingulo-Opercular-55_L-Ctx	MCC L	0.0758	0.6867	0.0783	0.5021
R_46	Cingulo-Opercular-14_R-Ctx	PRE R	0.0703	0.4085		
L_PFm	Frontoparietal-48_L-Ctx	IPG L	0.0687	0.1079		
R_PFm	Frontoparietal-24_R-Ctx	IPG R	0.0684	0.1450		
R_V3	Visual2-04_R-Ctx	MOG R	0.0624	0.3875	0.0512	0.2402
L_PF	Cingulo-Opercular-51_L-Ctx	SMG L	0.0595	0.2324		
R_LIPv	Visual2-16_R-Ctx	IOG R	0.0573	0.4676		
L_LIPd	Dorsal-Attention-15_L-Ctx	SPG L	0.0565	0.5653	0.0269	0.6293
R_TE1m	Frontoparietal-28_R-Ctx	MTG R			0.0834	0.1299
R_PH	Visual2-18_R-Ctx	IOG R			0.0613	0.5795
	Cingulo-Opercular-27_R-DCN	DCN R			0.0300	0.1277
R_d23ab	Default-05_R-Ctx	PCUN R			0.0294	0.3565
	Somatomotor-17_L-HIPP	HIPP L			0.0288	0.1443
L_a24	Default-44_L-Ctx	ACC L			0.0287	0.5909
L_p24	Cingulo-Opercular-56_L-Ctx	ACC L			0.0250	0.2293
R_a9-46v	Frontoparietal-13_R-Ctx	IFG R			0.0247	0.2629
R_V3A	Visual2-07_R-Ctx	MOG R			0.0219	0.0424
	Somatomotor-21_R-HIPP	HIPP R			0.0216	0.2283
R_8Av	Default-11_R-Ctx	PCUN R			0.0190	0.2532
R_IP2	Frontoparietal-22_R-Ctx	IPG R			0.0183	0.5670
